# Interrogation of Oxidative Pulsed Methods for the Stabilization of Copper Electrodes for CO_2_ Electrolysis

**DOI:** 10.1021/jacs.4c06284

**Published:** 2024-07-05

**Authors:** Jesse Kok, Jim de Ruiter, Ward van der Stam, Thomas Burdyny

**Affiliations:** † Materials for Energy Conversion and Storage (MECS), Department of Chemical Engineering, Faculty of Applied Sciences, Delft University of Technology, van der Maasweg 9, Delft, 2629 HZ, The Netherlands; ‡ Inorganic Chemistry and Catalysis, Debye Institute for Nanomaterials Science & Institute for Sustainable and Circular Chemistry, Utrecht University, Universiteitsweg 99, Utrecht, 3584 CG, The Netherlands

## Abstract

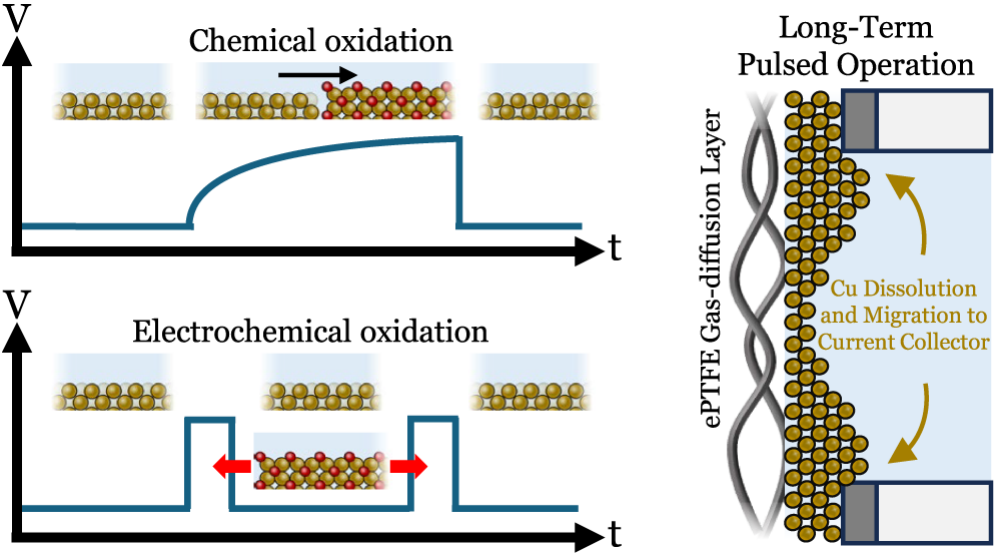

Using copper (Cu) as an electrocatalyst uniquely produces multicarbon products (C_2+_-products) during the CO_2_ reduction reaction (CO2RR). However, the CO2RR stability of Cu is presently 3 orders of magnitude shorter than required for commercial operation. One means of substantially increasing Cu catalyst lifetimes is through periodic oxidative processes, such as cathodic–anodic pulsing. Despite 100-fold improvements, these oxidative methods only delay, but do not circumvent, degradation. Here, we provide an interrogation of chemical and electrochemical Cu oxidative processes to identify the mechanistic processes leading to stable CO2RR through electrochemical and *in situ* Raman spectroscopy measurements. We first examine chemical oxidation using an open-circuit potential (OCP), identifying that copper oxidation is regulated by the transient behavior of the OCP curve and limited by the rate of the oxygen reduction reaction (ORR). Increasing O_2_ flux to the cathode subsequently increased ORR rates, both extending lifetimes and reducing “off” times by 3-fold. In a separate approach, the formation of Cu_2_O is achieved through electrochemical oxidation. Here, we establish the minimum electrode potentials required for fast Cu oxidation (−0.28 V *vs* Ag/AgCl, 1 M KHCO_3_) by accounting for transient local pH changes and tracking oxidation charge transfer. Lastly, we performed a stability test resulting in a 20-fold increase in stable ethylene production *versus* the continuous case, finding that spatial copper migration is slowed but not mitigated by oxidative pulsing approaches alone.

## Introduction

With the use of electrical energy as an input, carbon dioxide (CO_2_) can be converted to useful chemical compounds, enabling a more circular industrial carbon cycle.^1^ One enabling technology is CO_2_ electrolyzers which can form a bridge between renewable energy sources and the production of chemical feedstocks. The product species found at the outlet of CO_2_ electrolyzers, however, depend on the cathode material used and the reaction medium.^2−4^ Using copper (Cu) allows for the formation of multicarbon (C_2+_) products, such as ethylene (C_2_H_4_), ethanol (C_2_H_5_OH), and acetate (CH_3_COOH).^5^ These products are desirable compounds given their market value and function in society.^6,7^

Electrochemical production methods, however, must be cost-competitive in order to flourish, placing an emphasis on performance metrics such as Cu product selectivity,^8−10^ efficiency, current density, and stability. Increases in current densities have been enabled through the introduction of gas diffusion electrodes (GDEs),^11−13^ while placing GDEs in a membrane electrode assembly (MEA)^14^ allows for high energy efficiencies. Unfortunately, Cu stability acts as a major barrier to the commercialization of CO_2_ electrolyzers, where operational time scales of >40,000 h of CO_2_ reduction reaction (CO2RR) production are needed.^15^ The Faradaic efficiency (FE) of C_2_H_4_ and other C_2_-products for most lab-scale systems currently degrades within 10 h of operation due to a mixture of Cu catalyst degradation,^16−18^ impurity deposition,^19,20^ and salt formation.^21,22^ Of these possible forms of failure mechanisms, the degradation of Cu is the most unresolved and severely hampers CO_2_ electrolyzers.

In recent years, the degradation of Cu catalysts in near neutral media has been investigated more intensively, with a specific focus on the mechanisms resulting in structural deformations, also called restructuring. These mechanisms have been separately identified as oxidation-induced, electrolyte-induced, potential-induced, and carbon monoxide (CO) induced. For example, in the work by Raaijman *et al.*, Cu restructuring was shown to be a result of the initial reduction step of the copper oxide (Cu_*x*_O) precatalyst to Cu upon applying a potential.^20^ Further, when a Cu electrode is immersed in an electrolyte at the open circuit potential (OCP), the dissolution of Cu species can occur as an initial oxide layer forms.^23,24^ These changes to the structural morphology were also observed using *in situ* atomic force microscopy.^18^ Use of reducing potentials has been shown to, besides the dissolution and redeposition mechanism, induce Ostwald ripening, a phenomenon characterized by the growth of secondary particles.^25^ This particle dissolution and coalescence mechanism is frequently described as the first restructuring mechanism found after an oxidation/reduction cycle. *In situ* Raman spectroscopy and fluorescence measurements have shown the partial dissolution of the Cu electrode to be in the form of a copper–carbonate hydroxide complex.^24,26−28^ The carbonate ions within this complex allow for the direct formation of CO at low cathodic overpotentials.

With time, a transition from dissolution and coalescence into Ostwald ripening^29,30^ leads to a broadening of the particle size distribution. The migration of dissolved copper complexes is made possible here due to a local alkaline environment, where the presence of local OH^–^ ions has been shown to induce autonomous oxidation/redeposition reactions involving the metallic Cu species.^31^ In addition, cathodic corrosion could explain the migration of dissolved Cu complexes, similar to platinum.^32^ This mechanism was found to be the main cause of the deteriorating catalyst performance during cathodic polarization on the Cu catalyst, in both argon and CO environments.^33^ Here, nanofragments were formed during the reduction reactions and resulted in an increased CO_bridge_/CO_atop_ (spatiotemporal-resolved *in situ* Raman spectroscopy) ratio that poisoned the catalyst surface. Without removal of poisoned sites *via* methods such as pulsing, the catalytic selectivity to the desired products degrades. Despite the many proposed ways in which copper dissolves and redeposits over time, there is a consensus in the field that Cu restructuring in general is responsible for the loss of CO_2_ reduction selectivity, especially to C_2+_ compounds.

Although surface restructuring can be the dominant catalyst degradation pathway, the other possible failure mechanisms should not be excluded immediately. When a system is predominantly suffering from flooding or salt formation, it can be easily tracked through ECSA calculations, changing the local CO_2_ concentration or simply by studying the GDE post-operation. Evaluating the possibility of impurity deposition is more challenging. Impurities originating from the system materials or electrolyte salts, such as Fe, Zn, or Pb, can be reduced on the cathode electrode during electrolysis. As a result, the system can start showing an increase in the activity for the hydrogen evolution reaction (HER) over CO2RR.^34^ This results in a selectivity change over time, although the characteristics of the changes are different than when Cu restructuring is described as the main cause of catalyst degradation. Studies that exclude impurity deposition as a possible degradation do frequently so by showing the purity of the electrode using X-ray photoelectron spectroscopy (XPS). It can be an excellent method to study the top 0.5–2 nm surface composition, but as the catalyst layer is often in the order of 100 nm, there is a likelihood that the metallic impurities are present beyond the 2 nm detection limit of XPS.^33^

Electrochemical systems suffering from limited stability caused by the deposition of metallic impurities during reductive phases frequently have a low geometric cathode area to catholyte volume ratio in combination with a low roughness factor of the cathode catalyst.^19^ These systems often show decay in the FEs of CO2RR products within 10 min of applying a reductive current or potential. Chemically purifying the electrolyte, for example with ethylenediaminetetraacetic acid (EDTA), has been shown to increase the catalyst lifetime in multiple works and can be an excellent way to show how much the catalyst lifetime is affected by present impurities.^34,35^ Only by detailed evaluation of the FE trends of the CO2RR products and of the Cu electrode structure post-operation, failure mechanisms can be characterized as a main or minor contributor to the limited catalyst lifetime at different time scales.

In efforts to counteract Cu restructuring and “reset” a Cu electrode system during operation, pulsed electrolysis has been put forward as a method to achieve stable CO_2_ reduction.^36−38^ During pulsed electrolysis, the applied Cu electrode voltage is periodically varied between CO_2_ reducing potentials and more anodic potentials capable of passivating Cu to Cu_*x*_O. The formation of an oxide layer in the more anodic pulse, for example cuprite (Cu_2_O), is shown to revive the electrocatalytic activity toward C_2_ product formation during the cathodic pulse.^38−40^ The reasons for C_2+_ product regeneration are heavily debated. For example, some works state that reducing the Cu_2_O to oxide-derived copper (OD-Cu) leads to increased density of grain boundaries and surface defects, promoting the formation of C_2_ products like C_2_H_5_OH or C_2_H_4_.^9^ Other works, such as the work from Xiao *et al.*, suggest that the promoted electrochemical activity of OD-Cu originates from simultaneously present metallic copper (Cu^0^) and oxidized copper (Cu^+^) under reducing conditions.^41,42^ However, these statements have frequently been discredited by other scientific works using electron energy loss spectroscopy and XPS as these show no Cu^+^ species to remain present during CO2RR. Notably, the thermodynamic energy barrier for the reduction of Cu^+^ to Cu^0^ is lower than that required to perform CO2RR, and beyond subsecond time scales at reducing potentials all oxide species should be reduced.^43,44^

As an added complexity, pulsed operation can be applied in numerous ways to form Cu passivation layers, with each approach impacting product selectivity and stability differently. Work from Obasanjo *et al.* set the Cu electrode as an anode, using oxidative currents to electrochemically form Cu_*x*_O.^36^ Conversely, Nguyen *et al.* utilized 15 min periods of OCP to achieve chemical oxidation of a catalyst deposited onto a GDE, which also renewed the cathode’s activity toward C_2_H_4_.^37^ In both of the above-described works, *in situ* Raman spectroscopy showed the formation of Cu_*x*_O when the cathode was exposed to oxidation charges or the OCP.

The improved stability was attributed to the formation of these oxides through both the electrochemical and chemical oxidation steps *via* pulsed operation, but the impacts of precise anodic potentials, the effects of local pH, and chemical oxidation rates can all be varied to give different copper stabilities.

More importantly, while pulsing has shown the ability to increase catalyst lifetimes, activity is still shown to steadily degrade over longer time scales. Thus, net restructuring is either still happening even with pulsed operation, or long operation reveals a previously uninvestigated separate degradation pathway. Greater knowledge and contextualization on the chemical processes involved in pulsed electrolysis for extending catalyst lifetime are a necessity to understand and extend Cu catalyst lifetimes.

Within this work, we provide context and the underlying chemical conditions governing copper oxidation processes during pulsed electrolysis to maintain the catalyst’s activity toward C_2_ product formation through a combination of electrochemical and *in situ* Raman spectroscopic measurements. We examine both chemical and electrochemical oxidation processes which generate Cu_2_O to formulate the different criteria necessary to extend C_2_H_4_ production from CO_2_ electrolysis. By examining oxidation potential, time, and charge, we define the half reactions occurring and the minimum required anodic pulse time. With the formulated criteria, a stability test was designed reaching 18 h of CO_2_ electrolysis with a minimal drop in the FE of C_2_H_4_ under realistic reaction conditions.

## Results and Discussion

In this work, for all experiments unless stated otherwise, we used a 300 nm Cu catalyst layer sputtered onto a polytetrafluoroethylene (PTFE) GDE and operated in a PEEK-flow cell with a flowing catholyte. Using the flowing catholyte and a PTFE GDE avoids both salt formation and flooding instabilities in all tests allowing for the catalyst stability to be assessed individually on the basis of Cu restructuring. Further, no pretreatments or roughening have been done to the sputtered Cu, which oxidizes to copper(II) oxide (CuO) after exposure to air after deposition. A 1 M KHCO_3_ catholyte was also chosen for these experiments as this represents a stable CO_2_ electrolyzer configuration as compared to alkaline electrolytes. Notably, Cu catalysts are significantly more stable in higher pH electrolytes, but the use of a neutral-pH electrolyte is more representative of future applications. The PTFE sputtered Cu GDE (Figure S1), and all the details of the setup are found in Figures S2 and S3.

**Figure 1:**
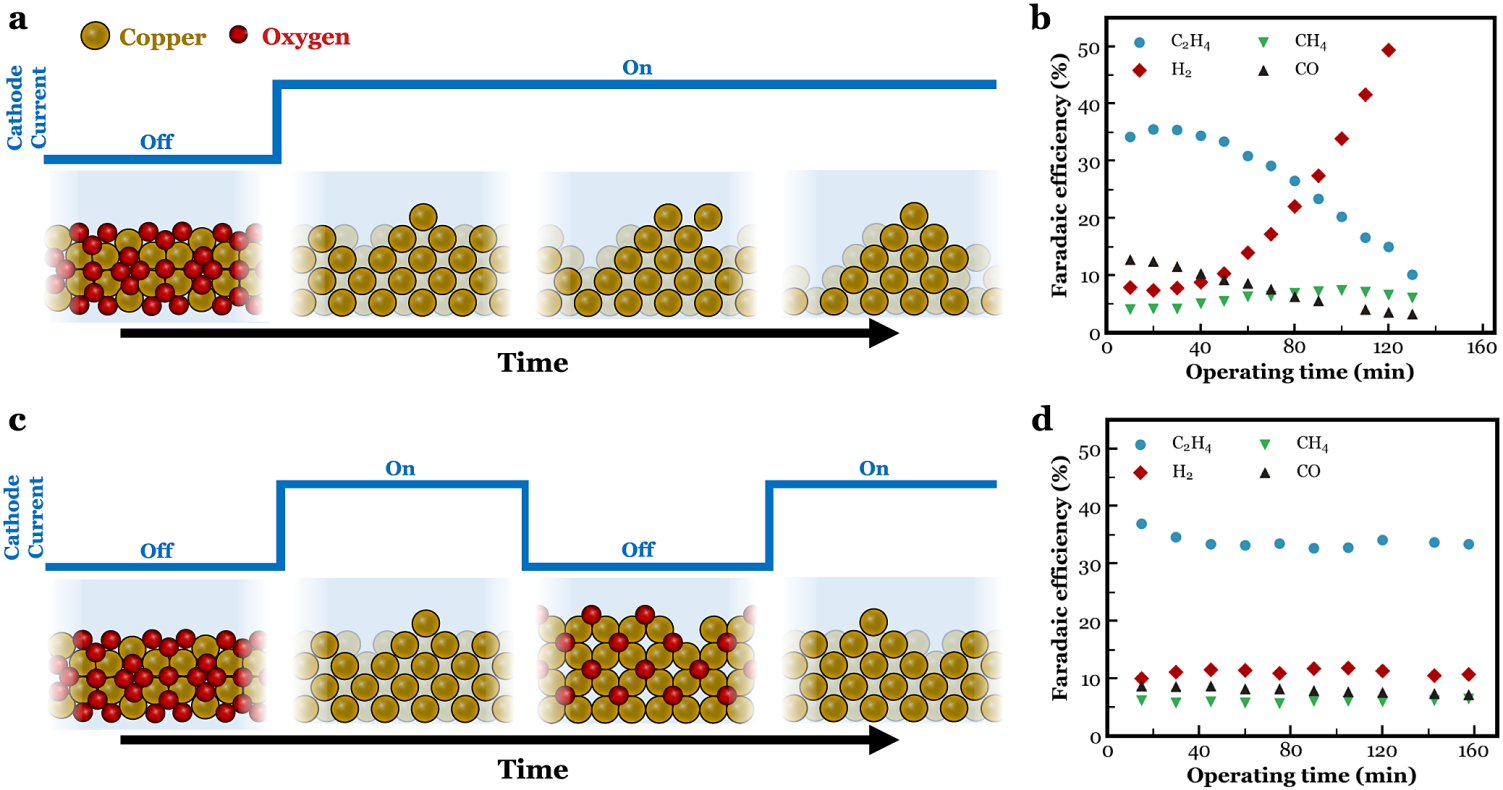
(a) Current profile during a constant chronopotentiometry experiment at −100 mA · cm^–2^ along with schematics illustrating the changes in chemical state and morphology at the different stages of the experiment. (b) FE curves of C_2_H_4_, CO, CH_4_, and H_2_ as a function of operating time during the experiment depicted in (a). (c) Current profile during pulsed CO_2_ electrolysis where a reductive current at −100 mA · cm^–2^ was alternated with an oxidative pulse. (d) FE curves of C_2_H_4_, CO, CH_4_, and H_2_ as a function of operating time during the experiment depicted in (c). Here, 15 min at −100 mA · cm^–2^ was followed by 15 min at OCP.

Using the above-described setup, we first wanted a continuous operation baseline (Figure 1a) with no pulsing as a benchmark to evaluate Cu stability. We chose a fixed current density of −100 mA·cm^–2^ and operated in chronopotentiometry mode, with the measured potential given in Figure S4. The gas FEs were measured during operation with a gas chromatograph (GC). Here, we will primarily use the C_2_H_4_ FE as a criterion for stability. While the selectivity of hydrogen (H_2_), methane (CH_4_), and CO also shows particular trends during degradation and adds further dimensions to the analysis, as the desired target product for the reaction, we will focus primarily on C_2_H_4_ as a stability descriptor. As shown in Figure 1b, while the C_2_H_4_ FE starts at 35%, there is a rapid decay within 1 h operation. Followed by an increase in H_2_ as was found to be consistent with other works.^36−38^ It is worth noting that these C_2_H_4_ FEs can likely be increased up to 40–50% by roughening, using higher current densities or a higher pH as shown elsewhere, but this was not a focus of the work. As a negative current was applied, the Cu is believed to initiate a restructuring response^45^ as the change in chemical state from Cu^+^ to Cu^0^ forces the material to take up a new unit cell. The high number of uncoordinated atoms at the surface can result in a significant amount of surface defects with this sudden change in the crystal lattice.^46^ The restructuring was studied using *ex situ* SEM analysis (see Figure S5). SEM images show a more agglomerative and dense structure at the sides of the GDE as well as large needlelike structures.^23,29,47,48^ The CO2RR gas product selectivity curves of three independent experiments continuously operated at current density of 100 mA · cm^–2^ are plotted in Figure S6. The time evolution of other products can provide additional information on the nature of the catalyst. After 1 h, the FE curves of C_2_H_4_ and CO experience a decay, whereas the activity of the Cu GDE toward CH_4_ and H_2_ increases. The increase in CH_4_ is especially more noticeable at a higher number of operational hours. Comparing Figures S2 and S5, it becomes clear that the electrode underwent a restructuring change as a result of early dissolution and redeposition and Ostwald ripening. With operating time, the spherical-like particles (Figure S2) were converted into smaller cubic structures (Figure S5a) at the center of the GDE. The perimeter of the GDE shows, in comparison to the center, a much more agglomerated dense structure (Figure S5b). A difference in Cu structure has been correlated with different CO2RR product activities.^49^

We speculate the difference in morphology between the center and perimeter of the Cu GDE to be the result of the uneven potential distribution across the GDE, resulting in directional migration of dissolved and redeposited species.

Looking at our continuous operation benchmark, we then wanted to confirm that other failure modes (impurity deposition, salt formation, and flooding) were not dominant on these time scales. Some conclusions can be taken from the selectivity trends displayed in Figure S6. As deposited metallic impurities should suppress the formation of all hydrocarbons, impurity deposition is not likely to be a dominating factor in the observed results. This is to be expected as the geometric area of the electrode–to–catholyte volume ratio of our electrochemical cell (2 cm^–1^), the roughness factor of the used GDE (>12) as determined by an ECSA analysis (Figure S7 and Table S2), and the time scale of the observed change in selectivity all suggest the impurity deposition to be a less plausible explanation of our results. As a confirmatory analysis, with energy-dispersive X-ray spectroscopy (EDS), no metallic impurities were found to accumulate on the catalyst surface within the detection limits of the technique (Figure S8 and Table S3).

Furthermore, no salt particles were found on the GDE after the test (Figure S9). Hence, the observed trend of product selectivity is attributed to the morphology and chemical state change of copper with operating time.

We also assessed the comparable rate of C_2_H_4_ decay as a function of catalyst layer thickness, applied current density, and catholyte pH (Figures S10–S12) all of which will impact the amount of Cu restructuring and the stability during operation. The results show a clear relation between catalyst layer thickness and stability, with thicker catalyst maintaining higher stability during operation. Although a higher current density proved to be detrimental in terms of stability for a thin Cu layer (100 nm), thicker catalyst appeared to show improved stability and starting FE of C_2_H_4_ with increasing current density in the first operational hour. These improvements in performance metrics are suspected to be a result of the increase in local pH with higher current densities as this is known to suppress HER.^50^ However, going beyond a certain current density does not pay back with improved stability. Similarly, utilizing a higher pH catholyte, for example 0.5 M K_2_CO_3_ (pH = 12), resulted in both a more stable and more selective C_2_H_4_ production as compared to the 1 M KHCO_3_ catholyte (pH = 8.3). Understanding the effect of the above evaluated parameters on the lifetime of Cu catalyst allows for the design of an experimental setup that can assess the stability in a low number of operational hours.

As discussed before, (electro)chemical oxidation can be periodically implemented to stabilize the FE of C_2_H_4_ and prevent the HER from becoming more dominant. A general illustration of these pulsed experiments is shown in Figure 1c. Here, we fixated the current density of the pulsed experiments at a reductive current density of −100 mA · cm^–2^, while varying the conditions of the oxidative pulse. During the oxidative pulse, the voltage on the Cu electrode is more anodic and can therefore result in the oxidation of Cu to Cu_2_O depending on the local pH and applied potential as prescribed by the Pourbaix diagram.^51^ An oxidative pulse can be done in two ways (i) either through setting the cathode to OCP resulting in chemical oxidation or (ii) by actively setting the Cu electrode as an anode and applying an oxidative current, resulting in electrochemical Cu oxidation. During pulsed operation, the length and potentials of the reductive pulses and oxidative pulses were controlled by a potentiostat. The effect of incorporating oxidation phases is shown in an initial experiment in Figure 1d. Here, a pulsed operation was applied where 15 min of operation at −100 mA · cm^–2^ (“on” time) was followed by 15 min of OCP (“off” time). These cycles then continued for a total CO_2_ reduction operating time (*e.g.*, time spend at −100 mA · cm^–2^) of 157 min. The potential during operation remained close to −2.2 V *vs* Ag/AgCl for both the continuous and pulsed operation in Figure 1 as shown in Figures S4 and S13. As shown in Figure 1d, a 3% drop in the FE of C_2_H_4_ was observed for the pulsed operation providing a stark contrast *versus* the continuous operation case (Figure 1a,b). These combined results then provide a baseline to further evaluate the effect of copper oxidation processes.

### Chemical Oxidation

With chemical oxidation established to extend Cu lifetimes as elsewhere,^37^ we sought to resolve the chemistry governing the oxidation process and the time-resolved growth of the oxide layers. In particular, we performed an electrochemical analysis of the Cu electrode under various “off” times to assess the importance of “off” time on Cu oxidation and C_2_H_4_ stability. Further, we examine how the addition of oxygen influences the cathode voltage during these “off” periods and cathode stability as this method has been previously utilized to lengthen catalyst lifetimes.^37^

**Figure 2:**
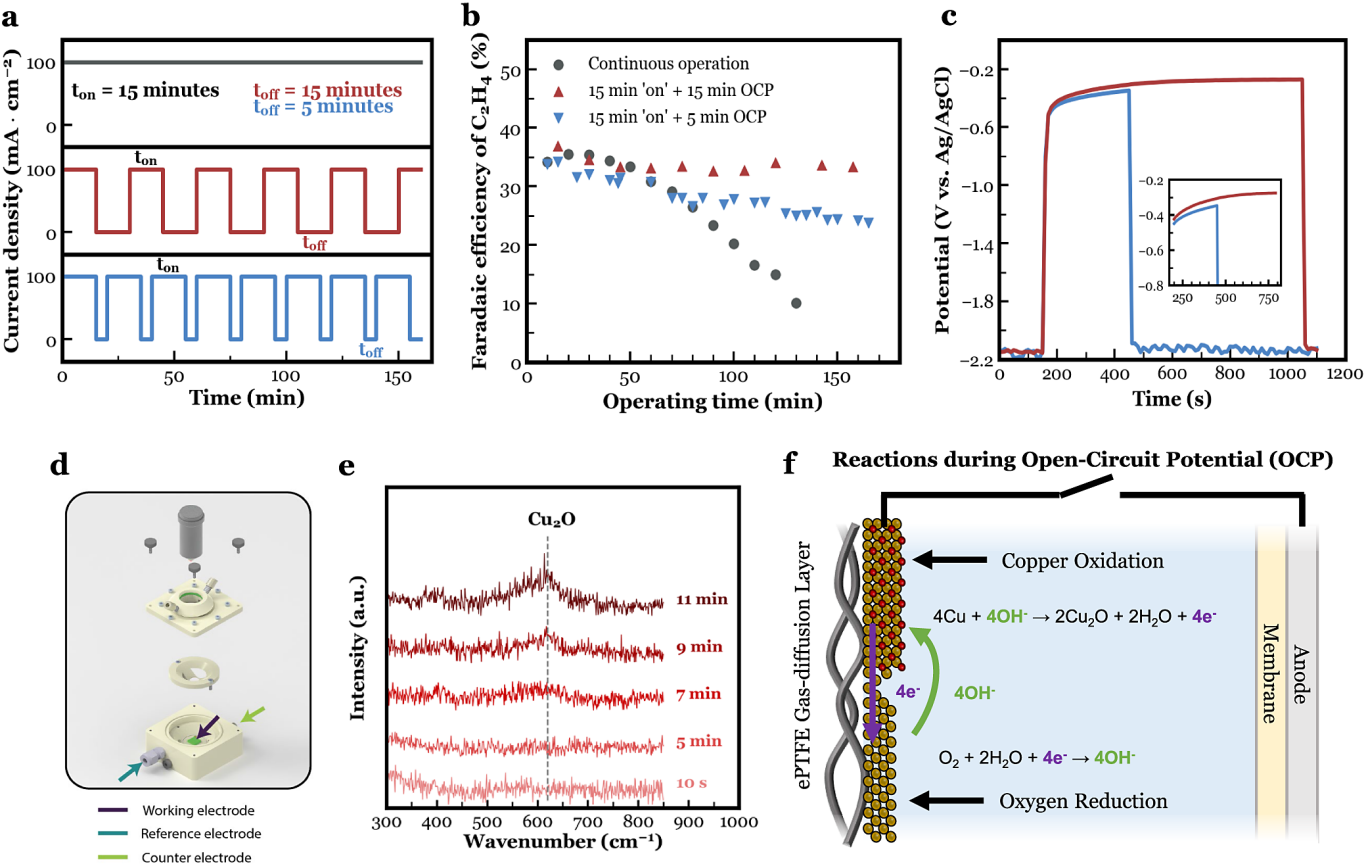
(a) The different performed chronopotentiometry experiments. A constant current density at −100 mA·cm^–2^ (black). A pulsed current with 15 min at −100 mA·cm^–2^, followed by 15 min at OCP (red). A pulsed current with 15 min at −100 mA·cm^–2^, followed by 5 min at OCP (blue). (b) FE of C_2_H_4_ as a function of time for the current profiles depicted in (a). (c) Change in OCP value with time during the oxidative pulse. (d) The *in situ* Raman spectroscopy setup. (e) Raman spectroscopy data showing the intensity of the Cu_2_O signal at different OCP times. (f) The half reactions associated with the formation of Cu_2_O during OCP.

As shown in Figure 2a, three scenarios were compared including continuous and pulsed operation, with OCP set during the anodic pulse. Here, the “on” times were fixed at 15 min, while the OCP periods were held at 15 and 5 min (Figures S13 and S14). When looking at the measured FE of C_2_H_4_ as a function of operating time in Figure 2b, we can see 5-min OCP periods are an improvement over the continuous case, but less stable than 15-min OCP periods which maintained most of the starting C_2_H_4_ FE after 157 min of operating time. The FEs of all the gas products are included in Figure S15. From these findings, it can be determined that chemical oxidation is a slow process, and that 5 min is insufficient to fully oxidize the entire Cu catalyst layer, although partial oxidation is occurring.

Despite the differences in the FE curves of C_2_H_4_ in Figure 2b, morphological changes can be observed to all three GDE samples as shown by the *ex situ* SEM images in Figures S5 and S16. The microneedles observed in the latter figure can be attributed to the formation of copper hydroxide (Cu(OH)_2_).^52,53^ These nanowires form as a consequence of Cu dissolving shortly before the passivation layer is formed.^48^

As confirmation that the OCP periods can also regenerate the activity of the Cu catalyst after the C_2_H_4_ selectivity decreased, we also performed experiments using longer reduction times of 1 h instead of the 15 min in Figure 2b. These results, shown in Figure S17, illustrate that a 15-min OCP period can recover catalyst activity.

To understand the relation between the programmed OCP time and Cu stability, we can look at the half reactions occurring on the cathode and track the electrochemical potential during OCP. There is no complete electrochemical circuit during OCP, meaning the cathode potential will equilibrate according to the electrode–electrolyte interface. Under these conditions upon turning the potentiostat off, the cathode potential will drop from the applied potential of −2.2 V *vs* Ag/AgCl to −0.5 V *vs* Ag/AgCl within 1 s (Figure 2c). This process is regulated by the capacitive discharge. After spending 15 min at OCP, the OCP approaches the thermodynamic value for a Cu electrode surface located in a 1 M KHCO_3_ solution (−0.27 V *vs* Ag/AgCl). The red curve (Figure 2c) stabilizes at this value, whereas spending only 5 min at OCP did not allow this to happen. Stabilization occurs close to a potential window where, as described by the Pourbaix diagram of our catalyst, Cu_2_O starts to become the thermodynamically most stable state.^51^ As 15 min at OCP allowed for more time to be spend in this potential window, more Cu is expected to become activated through oxidation/reduction cycles as compared to spending 5 min at OCP. Accordingly, the latter resulted in a mixed active/inactive CO_2_ electrocatalyst as indicated by the steadily decaying FE of C_2_H_4_ over time (Figure 2b), but one that is still more stable than the continuous case.

*In situ* Raman spectroscopy was then used to understand the correlation between the plotted OCP curves in Figure 2c and the catalyst lifetime as depicted in Figure 2b. The *in situ* Raman spectroscopy setup used for this purpose is shown in Figure 2d. The setup is explained in more detail in Supporting Information (see In Situ Raman Spectroscopy section). The Cu electrode used to perform Raman spectroscopy measurements differed from those used during electrochemical measurements. Formation of Cu_2_O was studied as a function of both potential and time by measuring Raman signals characteristic for the presence of Cu_2_O. Figure 2e shows a Raman signal at a wavenumber of 630 cm^–1^, one that can be assigned to Cu_2_O, after roughly 9 min of “off” time, which can be assigned to Cu_2_O. In literature, Raman spectra of Cu_2_O electrodes display three distinct features at 390, 520, and 630 cm^–1^, ascribed to (partially) Raman-allowed lattice vibrations.^28^ The 630 cm^–1^ feature (ascribed to defects in the Cu_2_O lattice) is the most intense vibrational band for Cu_2_O, and the signal-to-noise ratio of the current measurements only allows identification of this characteristic Cu_2_O band. The Raman data together with the OCP curves explain that the oxidation of Cu to Cu_2_O is not initiated immediately after switching from a reductive current density to OCP. Instead, some time is required for the properties of the local environment to equilibrate back to the bulk environment. This change then allows for the OCP to transient back to a potential that falls within the borders of the Cu_2_O region of the Pourbaix diagram.^51^ In other words, the fraction of the OCP period during which the chemical oxidation of Cu is set to take place is largely determined by the transient behavior of the OCP curve.

Within a certain OCP potential range of Figure 2c, Cu oxidation is then continuously occurring (eq 1 below). This oxidation requires sufficient time and is expected to require an oxygen source in the form of hydroxide. These hydroxides conveniently come from the oxygen reduction reaction (ORR) which also occurs during the OCP period and is given by eq 2. Although water reduction could also act as a supplier of these hydroxides, we decided to focus on the reaction that is thermodynamically the most favorable to occur, which is ORR. ORR requires electrons, which are no longer provided by the anode during open-circuit operation. The necessary electron exchange then takes place across the cathode/electrolyte interface where the electrons are provided by the Cu oxidation reaction. Thus, the Cu electrode has simultaneous oxygen reduction and Cu oxidation spatially occurring on the same electrode when oxygen is present in the electrolyte (Figure 2f), and over time will result in full oxidation of Cu as long as sufficient oxygen is available. The process then becomes self-limiting once Cu is fully passivated or oxygen is depleted.

The two half reactions responsible for passivating the Cu electrode, along with the total reaction, are shown in eqs 1–3.^54−56^
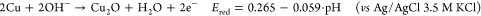
1
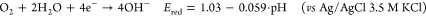
2
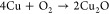
3

We note that the equations above are formulated for neutral and alkaline media specifically. In more acidic environments, the half reactions exchange water molecules and electrons, not hydroxides.

Prior to Cu_2_O formation, Cu dissolves at OCP.^29^ Using ICP-MS, it was found that for every minute at OCP, 0.5% of the initial present mass is lost due to dissolution (Table S4). Thus, an increased number of oxidation and reduction cycles and increased time spent at OCP lead to more structural deformations that impact stable C_2_H_4_ production.^20^ A sufficient amount of dissolved Cu is necessary before Cu_2_O becomes the thermodynamically most stable state at the bulk pH (8.3) as given by the Pourbaix diagrams for different local Cu concentrations.^51^

**Figure 3:**
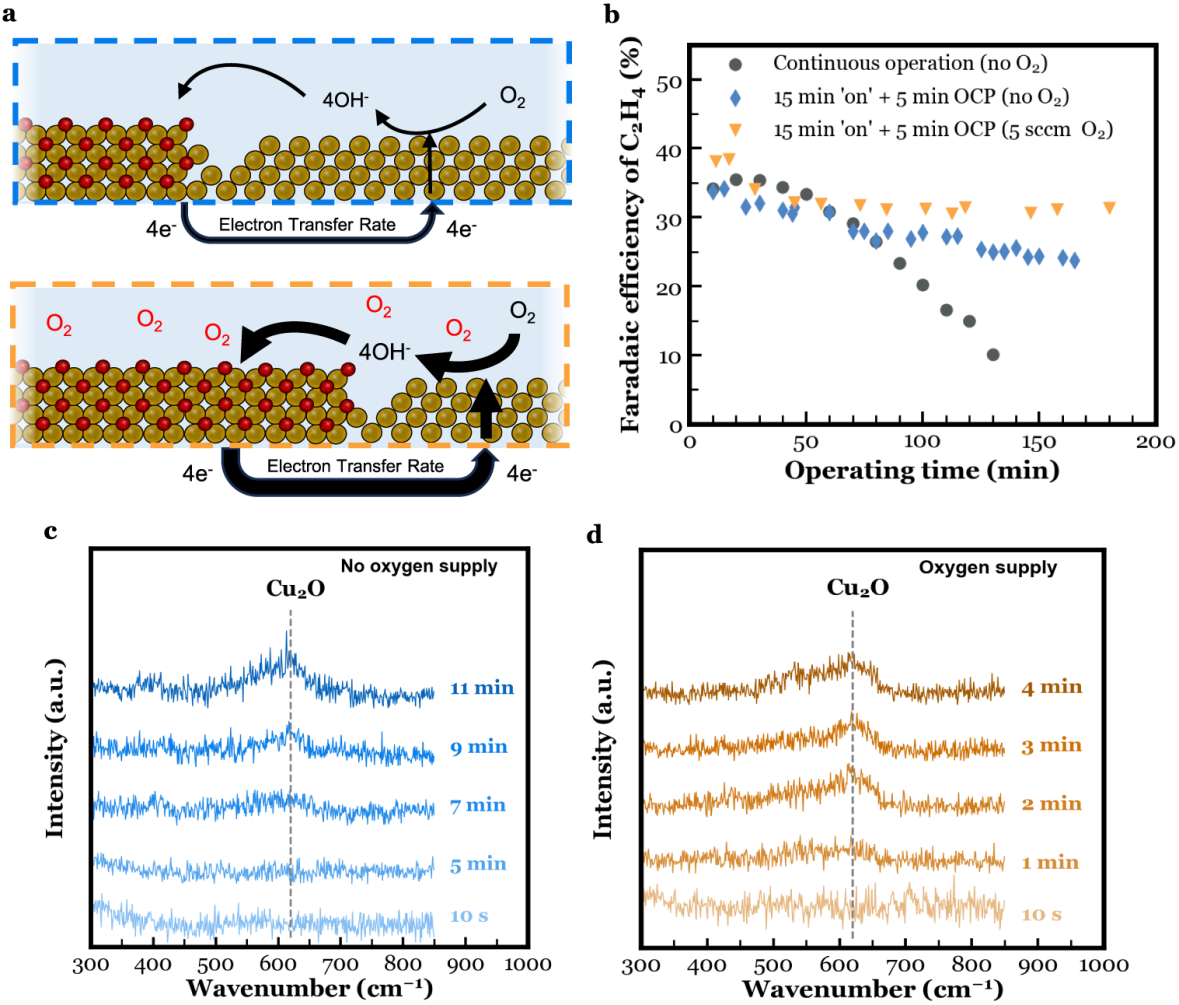
(a) A schematic illustrating the electron transfer between the copper oxidation reaction and the oxygen reduction reaction across the Cu electrode/electrolyte interface. The effect of a low (blue) or high (orange) oxygen flux to the cathode on the rate of the Cu_2_O formation is shown. (b) FE of C_2_H_4_ as a function of operating time for a continuous operation and the two pulsed electrolysis experiments shown in (a). Raman signal plotted between 300 and 850 cm^–1^ at different OCP times without (c) and with (d) added oxygen supply.

To reduce time spent at OCP, it is desired to accelerate full Cu oxidation. Given the hydroxide ion and electron dependency between the two half reactions, the reaction rates of both processes can be controlled by the supply of reactants (Figure 3a). The change in the rate of Cu_2_O formation and the OCP value can then be understood on a fundamental level using Evans (or mixed potential) diagram.^51,57^

Specifically, Cu_2_O formation is a corrosion process and can be described by the intersection of the anodic and cathodic polarization curves of the reactions in eqs 1 and 2, respectively, inside an Evans diagram. The construction of an Evans diagram can help understand the effect of reactant supply to the cathode on the chemical oxidation kinetics. We produced an anodic polarization curve of the Cu electrode by performing a linear sweep voltammetry from −0.4 to 0.3 V *vs* OCP at 20 mV/s (Figure S18). This potential sweep was chosen to first remove the already existing oxide layer. Cu oxidation then initiates at −0.28 V *vs* Ag/AgCl, and a maximum oxidation current is reached at 0.0 V *vs* Ag/AgCl. The 15-min OCP curve starts to flatten out near −0.28 V *vs* Ag/AgCl, further confirming the dependency of chemical oxidation on the transient behavior of the OCP curve. The expected cathodic polarization curve of the ORR is studied from literature and is expected to look similar to that depicted in Figure S19. Produced oxygen at the anode side that crossed over the membrane to the cathode and the little oxygen content of the catholyte are both the sources of the oxygen consumed by eq 2.

Figure S20 then shows the expected Evans diagram extracted from Figures S18 and S19. In Figure S21, the effect of increasing the oxygen content is visualized. The red line represents an anodic polarization curve for Cu oxidation, while the cathodic polarization curves are shown for a “low” (blue line) and “high” (yellow line) cathode oxygen content scenario. The plateau in the cathodic polarization curve is the limiting current density and is dependent on the oxygen availability (Figure S19). Thus, a greater oxygen presence will shift the cathodic polarization curve up and cause the intersection point of the ORR and Cu oxidation to move to higher rates (*y*-axis coordinate) and a more positive OCP (*x*-axis coordinate).^57−59^ With this positive shift in OCP, increased oxygen pressure in the cathode compartment allows the OCP after a reductive current density to transient back quicker to a potential at which Cu_2_O formation is allowed (Figure S18). With this in mind, the potential at which Cu starts to chemically oxidize to Cu_2_O is theoretically met faster with the increased oxygen pressure.

We can then conclude that the amount of reactivated Cu in a given anodic time frame is limited by the O_2_ content in the cathode compartment (Figure S20), which likely stems from crossover from the anolyte chamber through the membrane. To better understand these effects and shorten the required OCP time, we then performed the 5-min OCP experiment again (Figure 2a), but this time with oxygen supplied to the catholyte’s headspace at a rate of 5 sccm throughout the entirety of the experiment (Figure S22).

As shown in Figure 3b, we can then see that under a supply of oxygen, a 5-min OCP period is then sufficient to maintain the FE of C_2_H_4_ as compared to the scenario without added O_2_ to the catholyte. After 40 min of CO2RR, the FE of C_2_H_4_ stabilized at 30% and remained close to this value for another 140 min of operation. Thus, the chemical oxidation process was accelerated with the additional oxygen supply, resulting in a higher amount of Cu_2_O after 5 min at OCP compared to when no additional oxygen was supplied. By observing the measured cathode potential over time during OCP (Figure S22c), we can see a quicker thermodynamic stabilization toward the passivated Cu_2_O potential of −0.27 V *vs* Ag/AgCl. This positive shift in OCP is also expected based of the insights from the Evans diagram as shown in Figure S21.

The accelerated process of chemical oxidation with an elevated oxygen content at the cathode is further confirmed with *in situ* Raman spectroscopy data in Figure 3c,d. Here, the Raman signal between 300 and 850 cm^–1^ is plotted for different OCP times· The spectroscopy data verify that higher oxygen content near the Cu GDE significantly reduces the minimal required OCP time to start oxidizing Cu to Cu_2_O. Then, the oxygen-filled Raman setup detected the presence of Cu_2_O after less than 2 min at OCP. This duration is substantially shorter than the comparable case without added oxygen (∼7 min). Figure S23 shows Raman spectroscopy heatmaps where the Raman signal intensity is plotted as a continuous function of both time and wavenumber.

From eqs 1 and 2, it is also apparent that the measured OCP value is local pH dependent. This further explains the direction of the curves in Figure 2c. Beyond the potential jump regulated by capacitive discharge, the OCP curve is a result of the local pH equilibrating back to that of the bulk value.^59^ With the local environment becoming less alkaline with time, the equilibrium potentials of eqs 1 and 2 become more positive. Accordingly, the *x*-coordinate intersection of the polarization curves, the OCP value, becomes more positive with time. The flattening of the OCP curve indicates the local pH is then equal to bulk pH. This, in combination with the previously results, suggests that the fraction of the OCP period during which the chemical oxidation of Cu is set to take place is largely determined by what rate the local pH drops back to the bulk value. The relation between OCP curve, local pH, and growth of Cu_2_O is also schematically explained in Figure S24 using *in situ* Raman spectroscopy data.

In order to further demonstrate the effect of the change in local pH to the measured OCP value, a 1-h CO2RR experiment was performed in 0.5 M K_2_CO_3_ catholyte, followed by 5 min at OCP while recording the potential as a function of time (Figure S25). A lower increase in local pH during CO2RR is expected to occur when using K_2_CO_3_ as compared to KHCO_3_, meaning equilibration to the bulk pH should occur notably quicker for the former electrolyte. The difference between local pH and bulk pH is then plotted as a function of OCP time for both electrolytes in Figure S26. Figures S25 and S26 show the flattening of the OCP curve to occur as the local pH approaches the bulk pH.

The observations made in this section provide insights in the chemical processes that take place during OCP, how OCP can be utilized to increase the catalyst lifetime and how the variance of different parameters can alter the required “off” time. This “off” time is aimed to be minimized to both increase the capacity factor of the overall process and to reduce the loss of catalyst mass through dissolution at OCP. Further, by monitoring the OCP, the progress of Cu oxidation can be tracked during each “off” period. Interestingly, the requirement for O_2_ as a source of Cu oxidation also has implications for the usefulness of this pulsing approach. For example, in larger-scale systems where oxygen crossover across the membrane is either limited or may have spatial variations, it may be hard to guarantee full periodic oxidation of Cu catalysts without substantial “off” periods. These complications motivate more active or controllable Cu oxidation processes such as electrochemical routes in the next section.

### Electrochemical Oxidation

A different means of oxidizing Cu is through the use of oxidative currents as a result of setting the Cu electrode as an anode and withdrawing electrons. As a closed electrochemical circuit exists, this method is an electrochemical oxidation approach, but similarly results in the formation of Cu_*x*_O just like with chemical oxidation. However, the closed electrochemical circuit removes the necessity of consuming the liberated electrons by the ORR. Similar to the previous section, the Cu catalysts can be cycled through reduction and oxidative pulses to periodically recover CO_2_ reduction selectivity toward products such as C_2_H_4_. Despite achieving the same goal of oxidizing Cu, the chemical and electrochemical oxidation processes will impact Cu restructuring and stability differently. Here, we contrast the electrochemical oxidative processes. As the previous section showed the number of oxidized Cu particles to be an important parameter in stabilizing CO_2_ reduction through varying the length of the OCP period and oxygen levels, the same was believed to uphold for electrochemical oxidation with regards to potential. To assess this, CO2RR was performed for an hour at a reductive current density of −100 mA·cm^–2^, followed by 30 s at various “off” potentials ranging from −0.27 to −0.31 V *vs* Ag/AgCl (Figure S27a). These potentials fall within the measured OCP range in the previous section. These cathodic and anodic cycles were repeated for a total operating time of 3 h. In these experiments, however, the flow of electrons between the anode and cathode is not restrictive, and the oxidative current on the Cu electrode can be measured using the potentiostat (Figure S27b).

**Figure 4:**
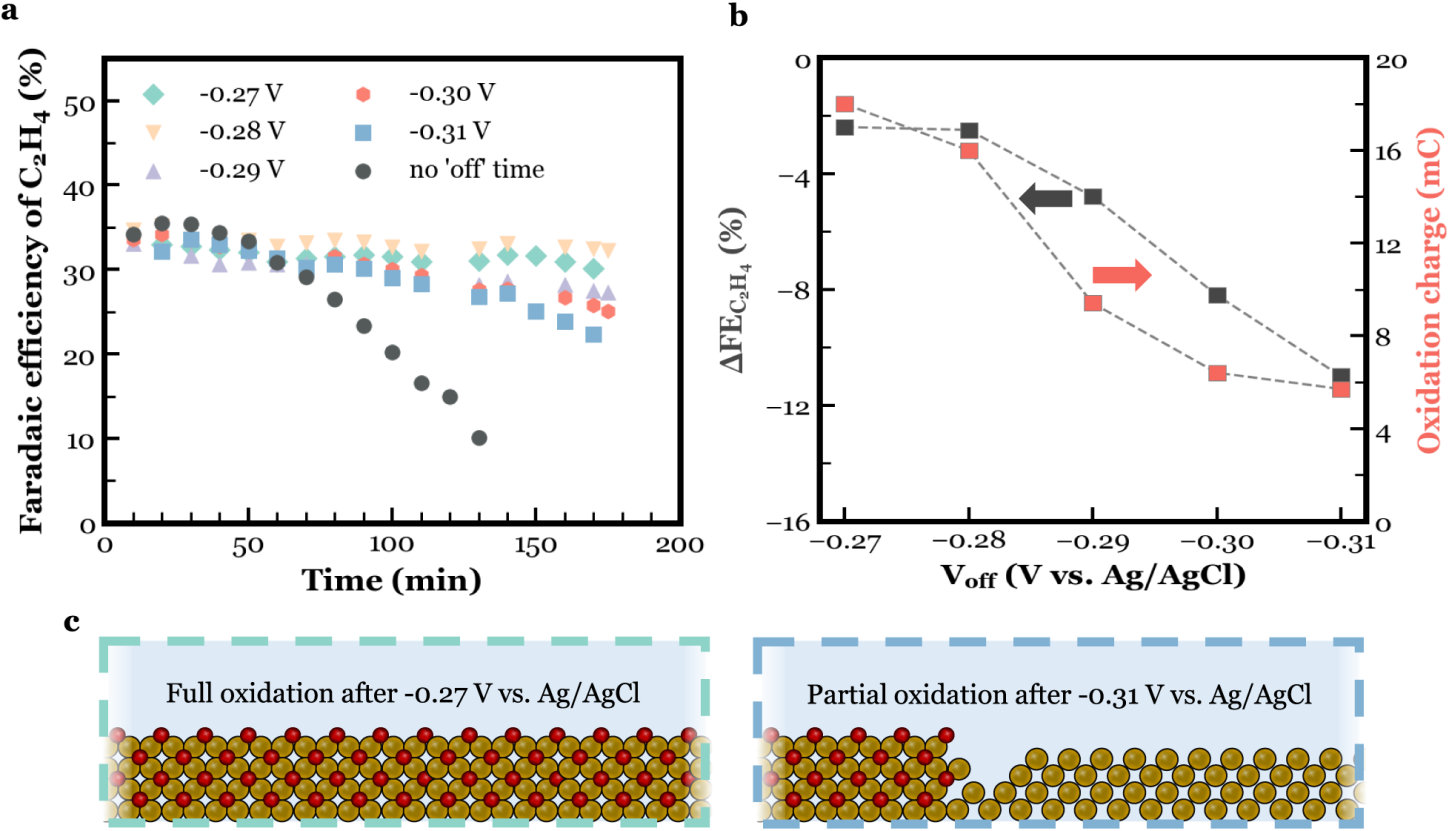
(a) The FE of C_2_H_4_ as a function of operating time for different “off” potential values (*vs* Ag/AgCl) during pulsed electrolysis. (b) The decay in the FE of C_2_H_4_ after 140 min of operating time (black) and the oxidation charge (red) as a function of programmed “off” potential. (c) Schematic picture showing the difference in Cu_2_O growth at two oxidation potentials.

The FE of C_2_H_4_ as a function of operating time for these various potential tests is shown in Figure 4a. Although only a minor difference of 10 mV between the oxidating potentials existed, differences in the effect on the catalyst lifetime were clearly visible (see Figure S28 for all gaseous products). In all cases, despite minor reduction in the ethylene FEs, a clear increase in H_2_ is seen in all cases after 1 h of operation. This indicates that for this pulsed approach, an operating time of 1 h using a 300 nm thick Cu layer is too long and will negatively impact CO2RR product formation overall.

More information can also be gained by observing the measured current during the electrochemical oxidation process at various potentials. As shown in Figure S27b, for each of the anodic potential steps there is a decaying current curve over time from which a sampled-current voltammogram can be derived.^60^ These current measurements can be analyzed to define the total accumulated charge (Figure 4b, red), which represents the total electrons withdrawn from the Cu electrode at each oxidation potential. When plotting the decay in the FE of C_2_H_4_ after 140 min of operation *versus* the total oxidative charge passed, there is a clear correlation (Figure 4b, black). These results indicate that a set potential of at least −0.28 V *vs* Ag/AgCl is required to sufficiently oxidize the entirety of the Cu electrode requiring a charge of roughly 16 mC for the electrode area and morphology. Potentials more negative than this value resulted in a lower FE of C_2_H_4_ after the three operational hours (Figure S27d). Interestingly, however, every “off” potential did result in a measured oxidation charge as shown in Figure 4c.

Potentially, similar to chemical oxidation, the degree to which the catalyst activity is maintained depends on the number of oxidized Cu particles during the oxidation periods. With the “off” potentials becoming more anodic, the measured oxidation charge increased, oxidation charge is proportional to the extent of passivation as illustrated in Figure 4d. Hence, the difference in the observed Cu catalyst lifetime with different applied oxidation potentials could be correlated, similar to chemical oxidation, to the degree to which Cu was oxidized.

Another possible reason for the detection of oxidation charge but a lack of C_2_H_4_ production stability with time would be the local pH effect on the stability of the different chemical states of Cu. Right after a reductive phase, the high local alkalinity would allow the Cu layer to oxidize even at potentials more cathodic than the −0.28 V *vs* Ag/AgCl which was based on the bulk pH. As the activity of CO2RR dropped to zero during this anodic phase and the oxidation reaction of Cu consumes OH^–^ ions, the local pH fell back to that of the bulk. With that in mind, during the 30 s “off” time, the catalyst layer could have moved from a condition in which Cu_2_O was stable, to metallic Cu being the more stable phase. The likelihood of this happening increases with more cathodic “off” potentials, as these are more near the borders of the Cu/Cu_2_O stability regions in the Pourbaix diagram anyway.

The approach of using chronoamperometry to oxidize your catalyst as opposed to chronopotentiometry, with the latter being the most frequently used method in other works, does come with the benefit of controlling your oxidation state more accurately. With chronopotentiometry, the lack of control of potential could result in the (partial) formation of CuO when Cu_2_O was the aimed compound.

In order to understand the effect of oxidative pulses on electrochemical surface area (ECSA), we utilized cyclic voltammetry to approximate the ECSA before and after continuous and pulsed electrolysis utilizing electrochemical oxidation. The analysis (Figure S7 and Table S2) shows no significant difference in the change of ECSA over time between the two types of operations, indicating that the observed stability difference is not driven by the surface area of the catalyst.^61^

The Raman data were visualized using a heatmap and are shown in Figure S29. The applied potential is plotted above the heatmap, showing the distinct reduction and oxidation phases.

### Stability Test

In the previous sections, pulsed electrolysis, both using chemical oxidation and electrochemical oxidation, was proven to lengthen the catalyst lifetime. Due to the choice of Cu catalyst layer thickness, catholyte pH, and current density, the effects of pulsed electrolysis could already be made visible in a small number of operational hours. In this section, pulsed electrolysis was used to perform a stability test. The changes made to the setup and materials to conduct the stability test are inSupporting Information.

**Figure 5:**
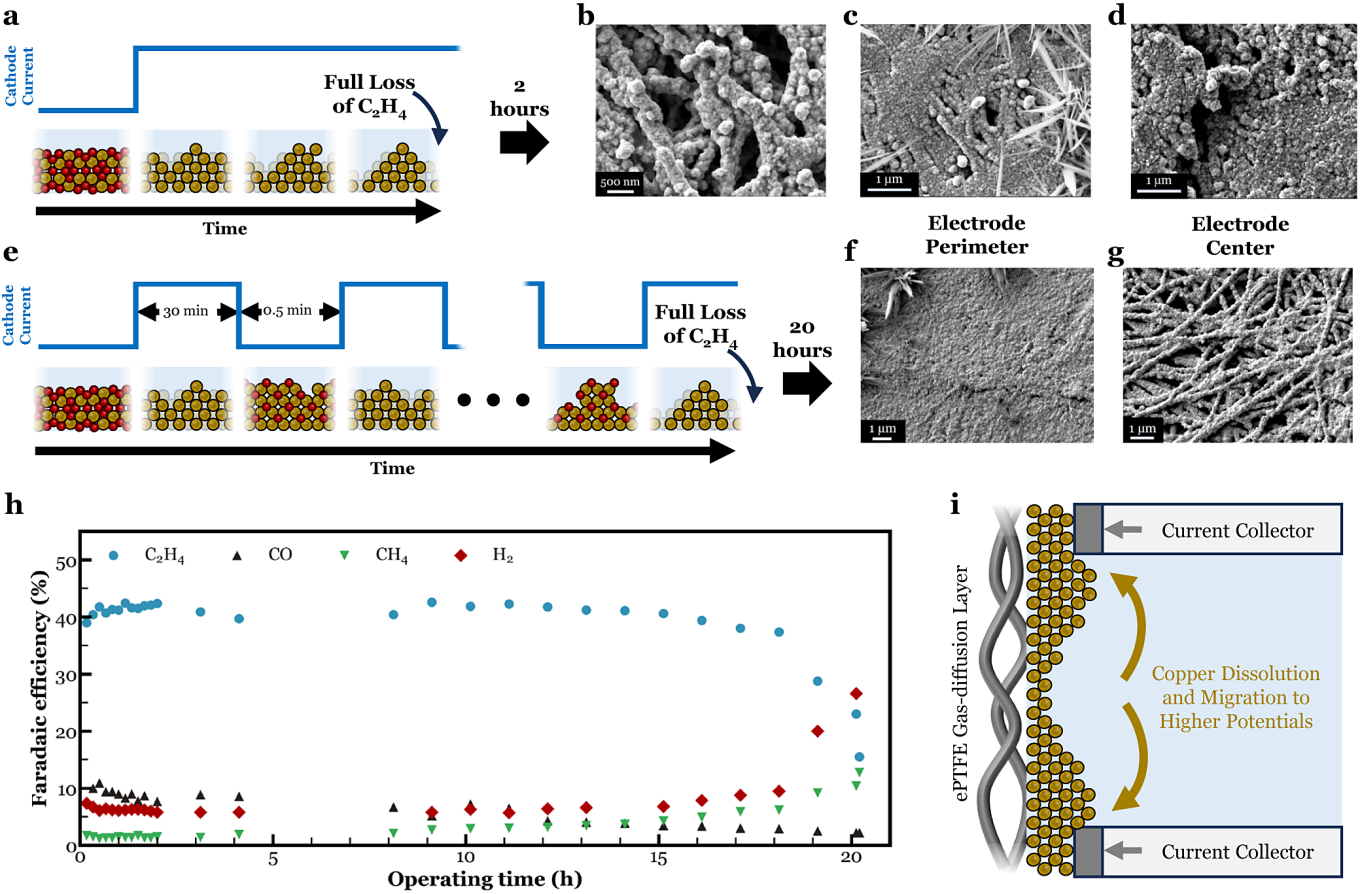
(a) Current profile during a constant chronopotentiometry experiment at −100 mA cm^–2^ along with schematics illustrating the changes in chemical state and morphology at the different stages of the experiment. (b–d) *Ex situ* postmortem SEM images of perimeter (b, 30,000× magnified and c, 25,000× magnified) and center (d, 25,000× magnified) of Cu GDE that was subjected to the current profile in (a). (e) Current profile during pulsed CO_2_ electrolysis where a 30-min reductive current at −100 mA cm^–2^ was followed by a 30 s oxidative pulse of 0.524 V *vs* RHE. (f,g) *Ex situ* postmortem SEM images of perimeter (f, 10,000× magnified) and center (g, 13,000× magnified) of Cu GDE that was subjected to the current profile in (d). (h) FE of gas products as a function of operating time during stability test. (i) Schematic illustrating the directional dissolution and migration as a result of the uneven potential distribution.

As of all the other results, the outcome of the stability test was compared to the continuous operation case. The current profile of the continuous operation along with the expected change in chemical state and morphology is shown in Figure 5a. SEM images of the perimeter and center of the Cu GDE that was subjected to the current profile in Figure 5a are shown in Figure 5b-d. The GDE’s perimeter shows a rougher more agglomerated surface consisting of nanofragments. To further illustrate the potential of maintaining catalyst activity toward C_2_H_4_, 30 min at −100 mA · cm^–2^ were followed by 30 s of oxidative pulses at 0.524 V *vs* RHE (Figure 5e). The resulting potential curve is shown in Figure S30. And although this approach delays Cu restructuring as Figure 5e suggests, Figure 5f,g still shows restructuring to have occurred. If one compares the center of the fresh as-sputtered Cu GDE in Figures S1 and 5f, it appears the center orientated copper particles have migrated to the external parts of the electrode, closest to the current collectors (Figure 5i). Literature suggests some dissolution, migration, and deposition occur during operation and in the oxidation/reduction process, resulting in material movement with each cycle.^20,48^ This migration of Cu can be overcome or limited through a variety of approaches, which are then expected to extend C_2_H_4_ further. For example, adding further Cu catalysts would prevent voltage disparities across the electrode,^62^ limiting preferential redeposition of Cu to the current collector regions. Additionally, preventing dissolved copper from entering the bulk electrolyte would limit movement per oxidation/reduction cycle. More *ex situ* SEM images are shown in Figure S31. The spatial migration of Cu due to voltage variations is also expected to worsen at elevated current densities as surface voltages would become even more uneven. The effect of aggravated restructuring at elevated current densities on the selectivity curves of CO2RR gas products has been previously and shown in Figure S11. We then stress that overcoming the observed failure mechanism is a first step to identifying and characterizing further mechanisms, and then facilitating higher current density stability tests.

Figure 5h shows the FE curves of the gas products with operating time. The full product distribution was retrieved at three distinguished operational times and can be found in Figure S32, along with a H NMR spectrum of the liquid products found in the catholyte in Figure S33. In the first operating hours, the Cu catalyst became more active toward C_2_H_4_. Potentially due to an increase in the number of active sites and performing chronopotentiometry at a fixed current density. The optimal current density for C_2_H_4_ can partially shift over time. After 18 h, the FE of C_2_H_4_ experiences a firm drop. Within 2 h, the FE of C_2_H_4_ dropped to 15%. Due to the extent of the restructuring, additional oxidation/reduction cycles would temporarily increase the C_2_H_4_ selectivity, but this was followed by a significant decay. Hence, it does not allow for the regeneration of the C_2_H_4_ selectivity back to former values.

With the time dependent selectivity of all gas products as a function of time plotted in Figure 5h, it is noteworthy to mention the decrease in CO production with time. The very same observation was made for the previously 3-h electrochemical pulsed operation tests as well. The steady decrease in CO could indicate a decrease in CO_2_ to CO catalytic sites, that then eventually shuts down C_2_-hydrocarbon production. Similarly, as CO decreases, H_2_ and CH_4_ increase, potentially implying that additional sites begin to favor these products. Previous works, for example, have shown that high or low ratios of Cu(100) to Cu(111) will promote C_2_H_4_ or CH_4_ and H_2_ formation, respectively, and the site distribution is known to change over time.^49^ Tracking products, like CO, may then also be used to detect early failure of the Cu catalyst. With lab tests moving into the 100s or 1000s hour range, such methodologies for early detection are important to increase throughput.

## Conclusion

The sustainable production of C_2_ hydrocarbons through CO_2_ electrolysis suffers from stability issues as Cu reconstructs and deactivates under a negative applied potential. In this work, we provided a deeper understanding of the chemical processes that occur during both chemical oxidation and electrochemical oxidation during pulsed electrolysis as a method to increase the lifetime of Cu catalysts for CO2RR. Specific system design choices allowed for a low number of operational hours to be sufficient for the study of the catalyst degradation. By varying the “off” time, the chemical oxidation of Cu to Cu_2_O was found to be kinetically limited by the oxygen flux to the cathode. This was confirmed by *in situ* Raman spectroscopy. Lengthening the catalyst lifetime using electrochemical oxidation was largely dependent on the programmed “off” potential. Here, the chosen “off” potentials, although only 10 mV apart, resulted in very different ethylene selectivity trends as a function of time. Although the closure of the electrochemical circuit during electrochemical oxidation changes the number of reactions involved, for both chemical and electrochemical oxidation it is the total reactivated area that is believed to play a key role in lengthening the catalyst lifetime. A stability test with implemented electrochemical oxidation periods achieved a stable FE of C_2_H_4_ of ∼38% for 18 h of operation. It appeared from the SEM images that the high number of oxidation/reduction cycles resulted in a large number of copper fragments dissolving and redepositing on the sides of the GDE. Looking into strategies to minimize this process is believed to hold great value for lengthening copper lifetimes during both pulsed and constant electrolysis.

## Data Availability

All data are made available in the manuscript and the Supporting Information. Raw data made available through deposition on the 4TU.Centre.

## Supporting Information Available

Details on the setup and materials used, SEM images, full product distribution of the stability test, Faradaic efficiency plots of all gas products, EDS data (PDF)
