# Supplementary material for: Interrogation of Oxidative Pulsed Methods for the Stabilization of Copper Electrodes for CO2 Electrolysis
Source: J Am Chem Soc. 2024 Jul 5;146(28):19509–20. doi: 10.1021/jacs.4c06284 (PMC11258781; doi:10.1021/jacs.4c06284)
Supplement: Supplementary file 1 — ja4c06284_si_001.pdf [file ja4c06284_si_001.pdf]

Supplementary information

Interrogation of oxidative pulsed methods for stabilization of copper electrodes for CO_2 electrolysis

Jesse Kok[1], Jim de Ruiter[2], Ward van der Stam[2], Thomas Burdyny[1*]

[1]Materials for Energy Conversion and Storage (MECS), Department of Chemical Engineering, Faculty of Applied Sciences, Delft University of Technology, van der Maasweg 9, 2629 HZ Delft, The Netherlands.

[2] Inorganic Chemistry and Catalysis, Debye Institute for Nanomaterials Science, Utrecht University, Universiteitsweg 99, 3584 CG Utrecht, The Netherlands,

*Corresponding author email: T.E.Burdyny@tudelft.nl

Method

The cathode gas diffusion electrode (GDE) consisted of an on PTFE 300 nm sputtered copper (Cu) (2.25 cm^2). The laminated polytetrafluorethylene (PTFE) membrane with a polypropene backbone (0.2 µm pore size with 25 µm layer thickness, Sterlitech) reduced the likelihood of flooding as it has hydrophobic properties.[1] The sputtering was performed using magnetic sputtering at 3 µbar of argon pressure with different sputtering times and/or sputter gun power to get the desired thickness. Two SEM images of a 300 nm sputtered Cu GDE are included in Fig. S1.

For all the experiments performed, a polyether ether ketone (PEEK) flow cell configuration was used. An image of the contents of the flow cell and a schematic overview of the setup is given by Figs. S2 and S3. The anolyte and catholyte consisted of 1 M KOH (Sigma Merck, 45 wt%) and 1 M $KHCO_3$ (Sigma Merck, 99.7%), respectively. The solutions were pumped into the flow cell compartments at a flow rate of 20 mL/min. A Nafion 115 membrane (Ion Power) was used to separate the two electrolytes. On the anode side, a nickel mesh catalyzed the oxygen evolution reaction. The cathode potentials were measured versus the Ag/AgCl 3.5 M KCl reference electrode (eDAQ). The bolts and nuts that close off the cell were tightened using a torque wrench set at 1.5 Nm. Both constant and pulsating currents were supplied by a Parstat4000 potentiostat. Regardless of the oxidative pulses, reduction currents were always set to -100 $\mathrm{mA \cdot cm^{-2}}$. The Faradaic efficiencies (FE) were calculated using the outlet gas concentrations measured by the gas chromatography (GC) unit in combination with the outlet flow rates measured by a mass flow meter (MFM). The inlet CO_2 flow rate was set at 40 sccm for every experiment using a mass flow controller (MFC). The calculation of the FE is discussed below.

For the stability test, the nickel mesh was replaced by a Ti IrOx/RuOx mesh (De Nora) to function as anode catalyst. As a consequence, the 1 M KOH anolyte had to be substituted with a 1 M $KHCO_3$ solution. In order to establish a higher number of operational hours, the Cu catalyst layer was thickened (500 nm). Finally, in order to circumvent any potential drift, which is a common feature of Ag/AgCl electrodes during long operations, a RHE electrode was used instead (Gaskatel). Liquid samples of both the anolyte and catholyte were taken after 1, 4 and 5 hours of operation. These were analyzed by a 600 MHz NMR instrument (Fig. S29). Maleic acid (Sigma Merck) was used as internal standard to compute the concentrations of the identified liquid products. The equation for calculating the FEs of liquid products is shown in Eq. S4.

FE calculations

For most of this work, only the selectivity of the gas products was considered. The concentration of component *x* (C_x) in the cathode outlet gas stream was measured using an online GC, taking injections every 5 minutes. The flow rate of the total outlet gas stream as a function of time was determined using a MFM (V_{total}). The FE of component x was calculated using Eq. S1

$$FE_x = \frac{C_x \cdot 10^{-6} \cdot V_{total} \cdot 10^{-6} \cdot \frac{P}{R \cdot T} \cdot n \cdot F}{i \cdot 60} \quad \text{(S1)}$$

Here, C_x was expressed in units of PPM (parts per million), the measured flow rate (V_{total}) in mL/min, the pressure (P) and temperature (T) were set to 101325 Pa and 273 K, respectively, n is the number of electrons consumed in the reduction reaction for the formation of product x, F is Faraday's constant (96485.3 C/mol) and i is the applied current in A.

The measured flow rate was corrected as the mass flow meter was calibrated for CO_2 only. This was done using a gas correction factor (Eqs. S2 and S3). The gas correction factors for the different chemical components present in the outlet gas stream are shown in Tab. S1.

$$V_{total,\ corrected} = \frac{V_{total,\ measured}}{0.74} \cdot C_{mix} \quad \text{(S2)}$$

$$C_{mix}^{-1} = \sum_{i=1}^{6} \frac{y_x}{\text{Gas correction factor}} \quad \text{(S3)}$$

Table S1. Gas correction factors for the different chemical components present in the cathode outlet stream.

Number compound	Name compound	Gas correction factor
1	Ethylene	0.6
2	Carbon monoxide	0.76
3	Methane	1
4	Hydrogen	1.01
5	Water	0.74
6	Carbon dioxide	0.79

$$FE_x = 100 \cdot \frac{\left(\frac{I_x}{I_{std}} \cdot \frac{N_{std}}{N_x} \ C_{std} \cdot \frac{V_{tube} \cdot V_{electrolyte}}{V_{sample}} \cdot n \cdot F\right)}{(i \cdot t)} \qquad \text{(S4)}$$

I_x = Integral component x
I_{std} = Integral internal standard
N_x = Number of protons component x
N_{std} = Number of protons internal standard
C_{std} = Concentration internal standard NMR tube (mol/L)
V_{tube} = Volume of content NMR tube (L)
V_{sample} = Volume of added catholyte/anolyte sample to NMR tube (L)
$V_{electrolyte}$ = Volume of catholyte/anolyte during experiment (L)
n = number of electrons consumed to produce liquid product x
F = Faraday's constant (96485.3 C/mol)
i = Current (A)
t = Time (s)

<u>Setup</u>

All experiments were completed using the very same setup and materials. The setup is schematically shown in Fig. S2. The PEEK-flow cell was connected to a Parstat4000 potentiostat. The available VersaStudio software was used to program the various applied current curves.

During operation, 40 sccm of CO_2 was led into the cathode gas channels by means of a MFC from Bronkhorst. The anolyte and catholyte were pumped into the anode and cathode compartment, respectively, at a flow rate of 20 mL/min using peristaltic pumps (Masterflex). The pressure buildup inside the catholyte and anolyte chambers were regulated using a BPR system (Bronkhorst) that was monitored and controlled through a LabView file. During the time in which current was applied to the PEEK-flow cell and CO_2 electrolysis was performed, the GC analyzed the contents of the cathode's outlet. These concentrations, along with the measured flow rates, were taken in order to calculate the FEs of the gas products as a function of time. A liquid trap was placed between the PEEK-flow cell and the GC to prevent water from entering the analytical instrument.

In situ **Raman spectroscopy**

The Raman spectroscopy measurements were performed on a Renishaw InVia Raman microscope with a 785 laser. For time-resolved measurements we measure in static mode with a Nikon N40X-NIR water-dipping objective, 2.5 mW laser power and an acquisition time of 780 ms that, together with mechanistic time-delay (e.g., open and closing of the shutter) resulted in a time resolution of 1 spectrum per second. Raman measurements with an extended wavenumber range, typically had a time resolution of ± 6 seconds per spectrum. This electrochemical Raman cell consists of a PEEK container with an operational volume of 5 mL electrolyte (0.1 M $KHCO_3$ or 1 M $KHCO_3$). From the side, the reference and counter electrode are fixed into the electrolyte. The working electrode, or catalyst, is mounted in the middle of the container, by pressing down a peek bar against a gold

pin. Electrical connection of the gold pin is accessed via the side of the container by a wire located underneath the container.

As a consequence of the Raman spectroscopy setup, a PTFE Cu GDE could not be used as this would not allow for sufficient electrical contact (PTFE is non conducting). Hence, as an alternative, Cu foil was used (0.127 mm, 99.9%, Alfa Aesar) with a 300 nm thick sputtered Cu layer in order to ensure enough roughness.

In order to evaluate the oxidation state of copper and the formation of copper-intermediate complexes during chemical oxidation and subsequent reduction, 10 minutes at -1.5 V vs. Ag/AgCl was followed with the open-circuit potential (OCP). The system was set to the OCP for as long as was necessary to clearly observe a Raman signal at 630 cm^{-1}, indicating the presence of Cu_2O. To

study the effect of oxygen on the rate of chemical oxidation, oxygen flow was directed through the setup at a rate of 20 mL/min.

Gaining mechanistic insights on the processes during electrochemical oxidation was done by alternating 1 minute periods of reducing potentials at -1.3 V vs. Ag/AgCl with 30 seconds at -0.065 V, -0.05 V and -0.03 V vs. Ag/AgCl.

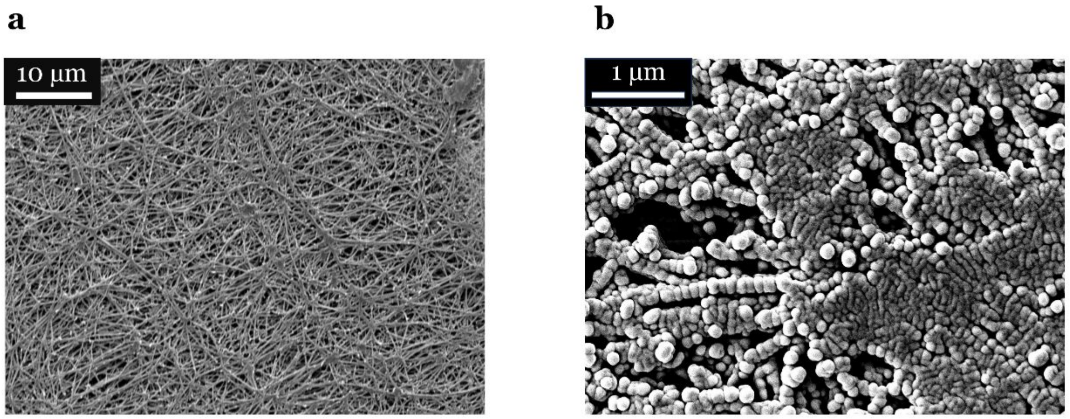

Figure S1. (a) A SEM image showing an on PTFE 300 nm sputtered layer of Cu (2000x magnified, E = 8.0 kV). (b) A SEM image showing an on PTFE 300 nm sputtered layer of Cu (25000x magnified, E = 5.0 kV).

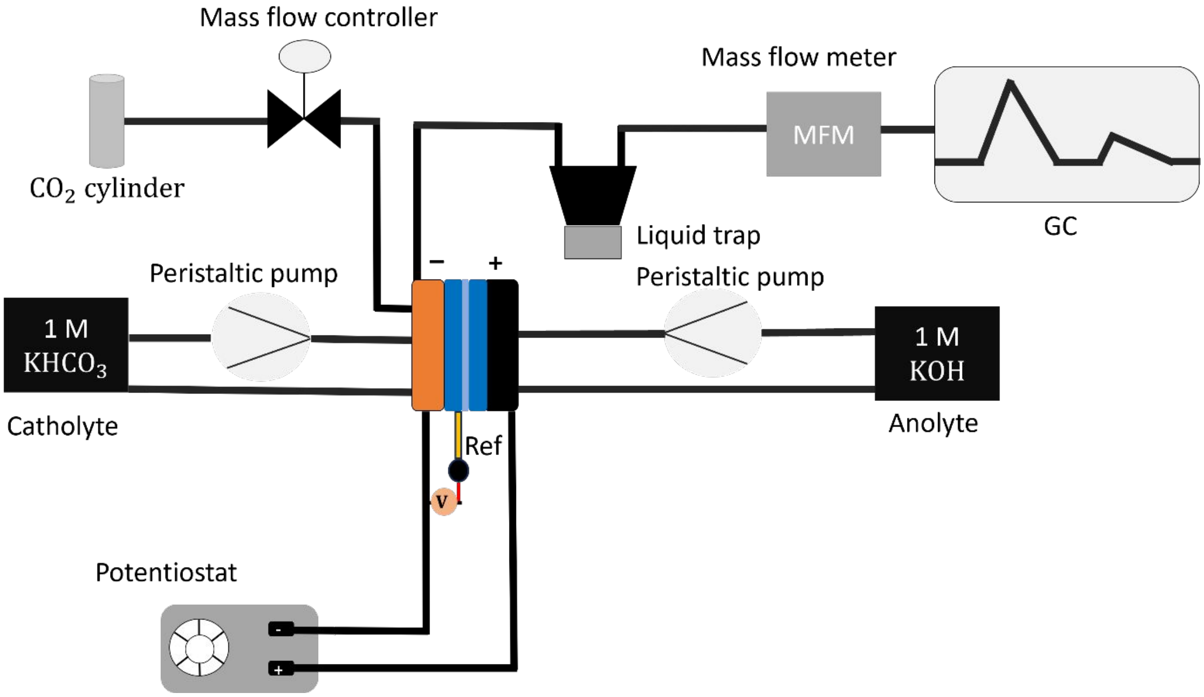

Figure S2. A schematic version of the setup used in the performed experiments.

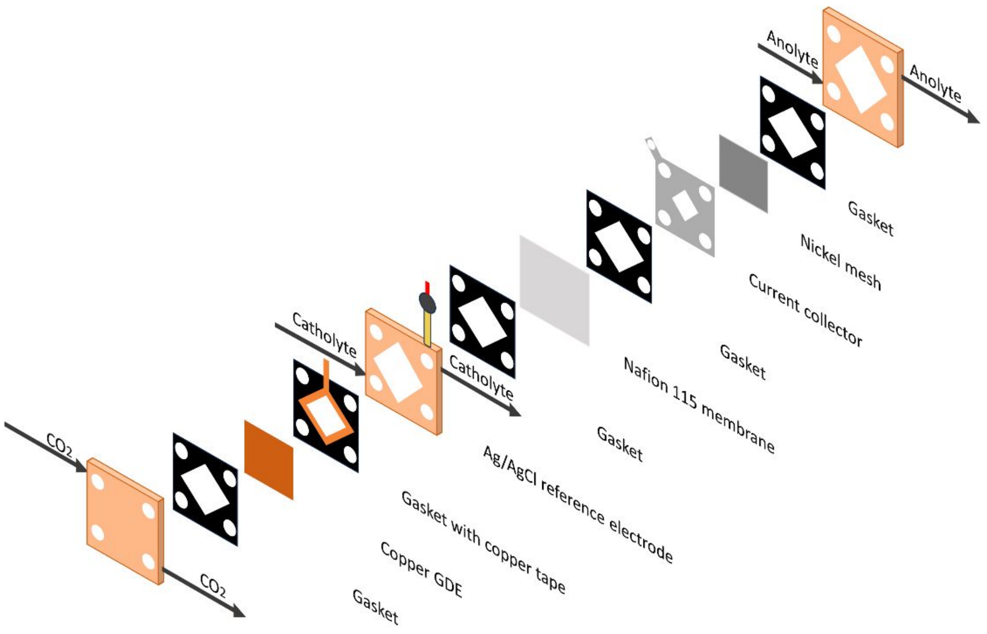

Figure S3. The different components making up the inside of the PEEK-flow cell.

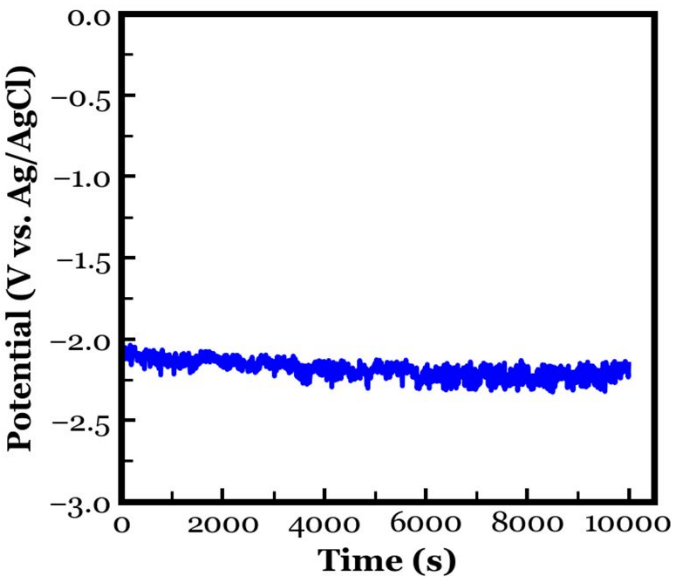

Figure S4. Potential (V vs. Ag/AgCl) without iR compensation as a function of time for a continuous operation at a current density of -100 mA · cm^{-2}.

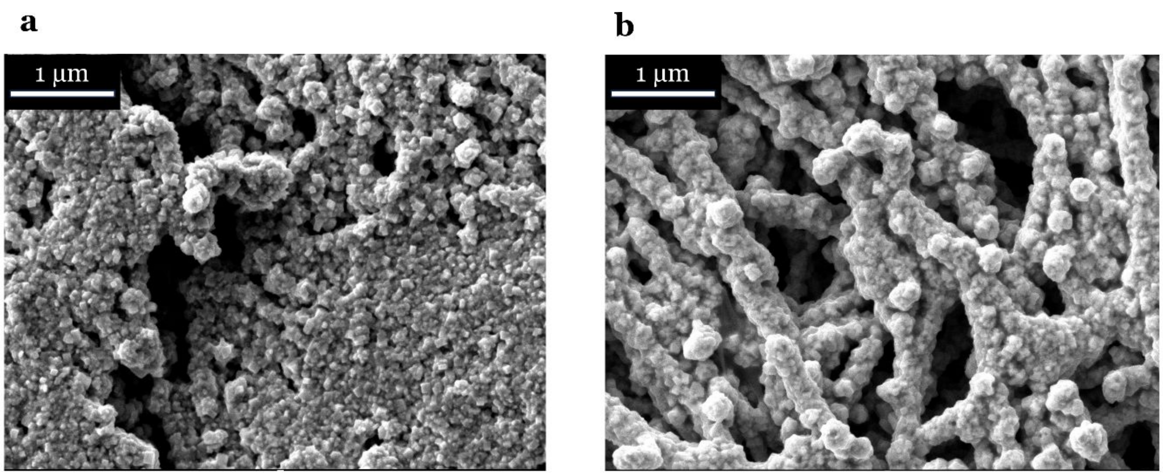

Figure S5. Post-mortem SEM images of a Cu GDE after 100 minutes continuous operation at a current density of -100 mA · cm^{-2}. (a) Center of Cu GDE (25000x magnified, E = 5.0 kV). (b) Perimeter of Cu GDE (25000x magnified, E = 5.0 kV).

Fig. S5a shows the damage done to the Cu GDE after a 100 minute exposure to a constant current density of -100 mA · cm^{-2}. From the same figure, it becomes evident that copper fragments migrated and agglomerated at the GDE perimeter. The concept of spatial activity in catalyst layers using PTFE as substrate was previously discussed by *Van Montfort et al.*[2]

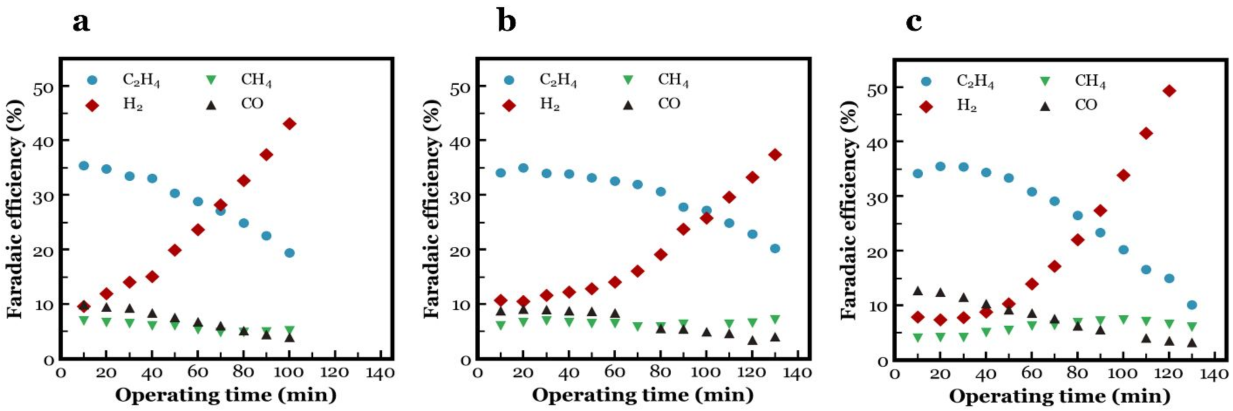

Figure S6. Graphs (a), (b) and (c) show the FEs of gas products for three separate experiments. Each graph represents a continuous operation at a current density of -100 mA · cm^{-2}.

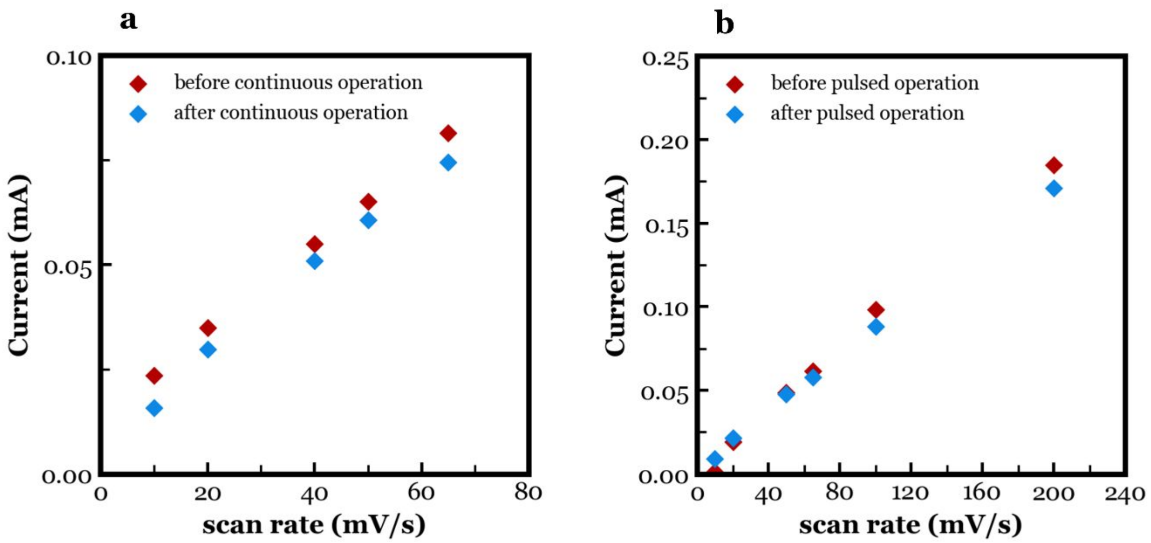

Figure S7. (a) Averaged current over cathodic and anodic sweep as a function of sweep rate before and after continuous operation. Extracted capacitance shown in Table S2. (b) Averaged current over cathodic and anodic sweep taken and plotted as a function of sweep rate before and after pulsed operation. Extracted capacitance shown in Tab. S2.

Table S2. Capacitance measurements done before and after a three hour long pulsed and continuous operation.

Type of operation	Before or after operation	Capacitance (mF/cm^2)
Continuous operation	Before	0.463
Continuous operation	After	0.422
Pulsed operation	Before	0.421
Pulsed operation	After	0.373

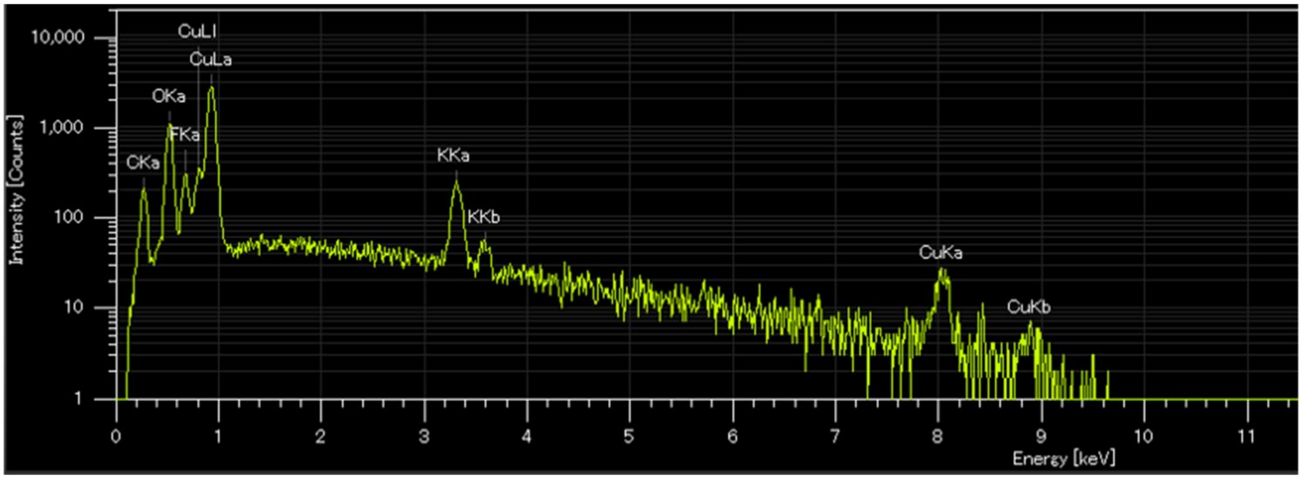

Figure S8. An energy dispersive X-ray spectroscopy (EDS) analysis performed on a Cu GDE sample after a continuous operation.

Table S3. Tabulated data of figure S7.

Element	Line	Mass%	Atom%
C	K	5.22 ± 0.12	14.21 ± 0.32
O	K	19.50 ± 0.32	39.86 ± 0.64
F	K	3.76 ± 0.14	6.47 ± 0.25
K	K	8.16 ± 0.31	6.83 ± 0.26
Cu	L	63.36 ± 0.72	32.62 ± 0.37

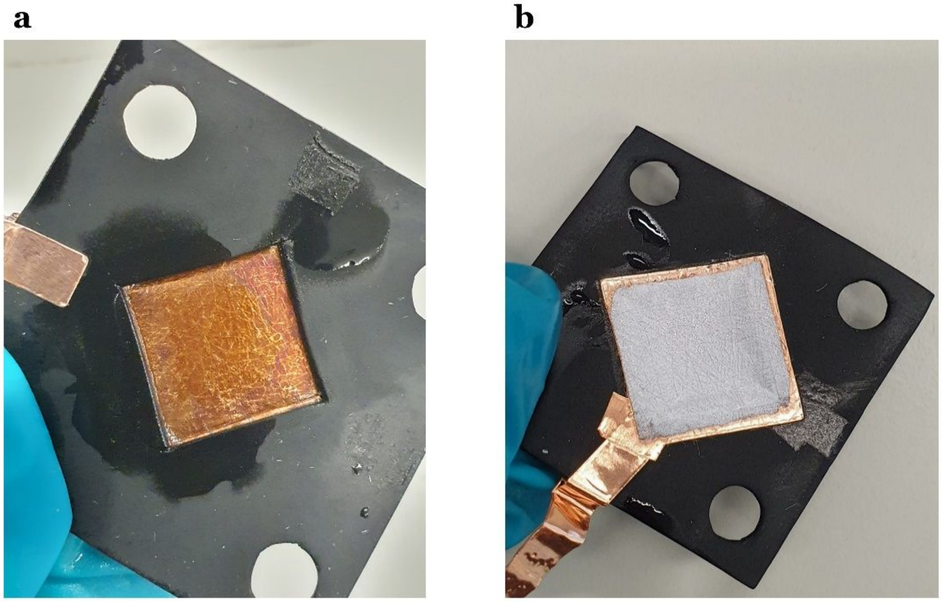

Figure S9. Cu GDE after being subjected to a continuous operation at -100 mA · cm^{-2} for 2 hour and 30 minutes. (a) Image front of GDE. (b) Image back of GDE.

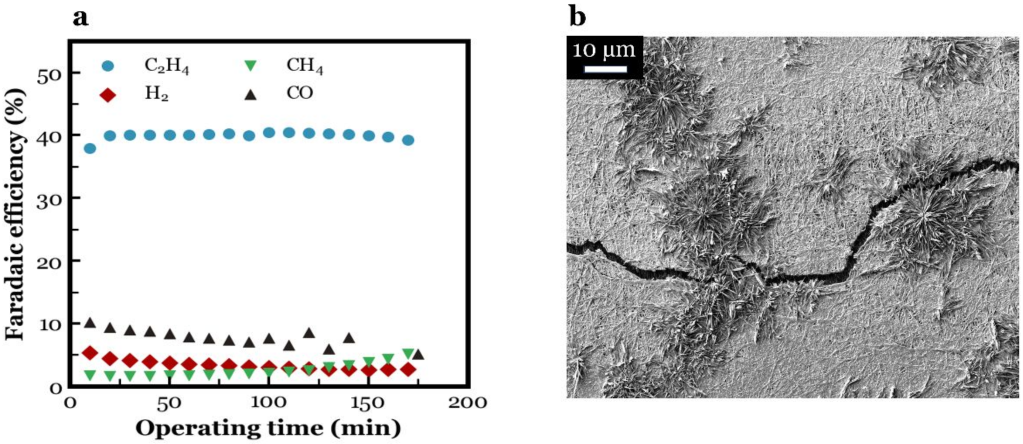

Figure S10. (a) FEs of gas products during continuous operation on a 300 nm Cu GDE at -100 mA · cm^{-2} in a 0.5 M K_2CO_3 (pH = 12.0) catholyte. (b) A SEM image showing the degradation on the perimeter of the Cu GDE after three hours of continuous operation using 0.5 M K_2CO_3 as catholyte (1000x magnified, E = 1.0 kV).

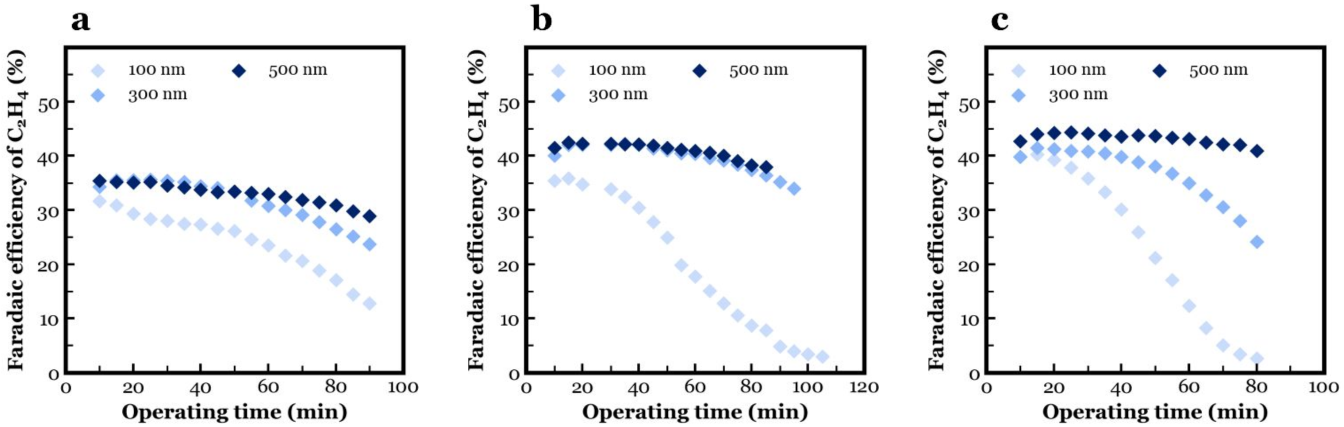

Figure S11. FE of ethylene as a function of operating time for different Cu catalyst layer thicknesses during a continuous operation at a current density of (a) -100 $mA \cdot cm^{-2}$, (b) -150 $mA \cdot cm^{-2}$ and (c) -200 $mA \cdot cm^{-2}$.

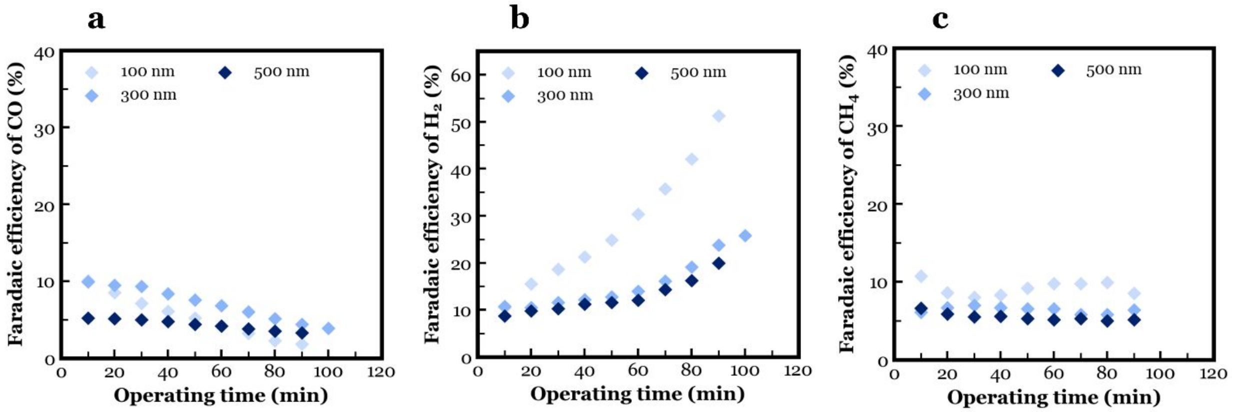

Figure S12. FE of (a) carbon monoxide, (b) hydrogen and (c) methane as a function of operating time during a continuous operation at a current density of -100 $mA \cdot cm^{-2}$ for different catalyst layer thicknesses.

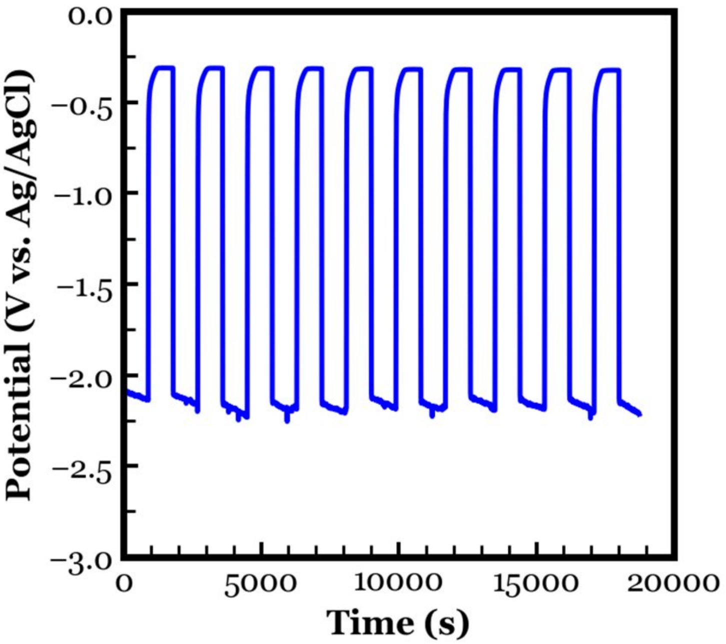

Figure S13. Potential (V vs. Ag/AgCl) without iR compensation as a function of time for a pulsed electrolysis experiment. 15 minutes at -100 mA · cm^{-2} was followed by 15 minutes at OCP.

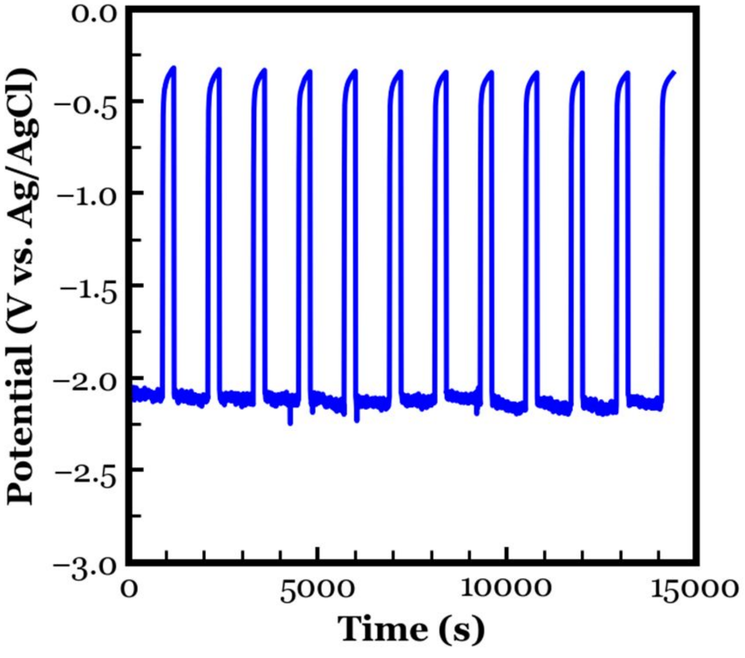

Figure S14. Potential (V vs. Ag/AgCl) without iR compensation as a function of time for a pulsed electrolysis experiment. 15 minutes at -100 mA · cm^{-2} was followed by 5 minutes at OCP.

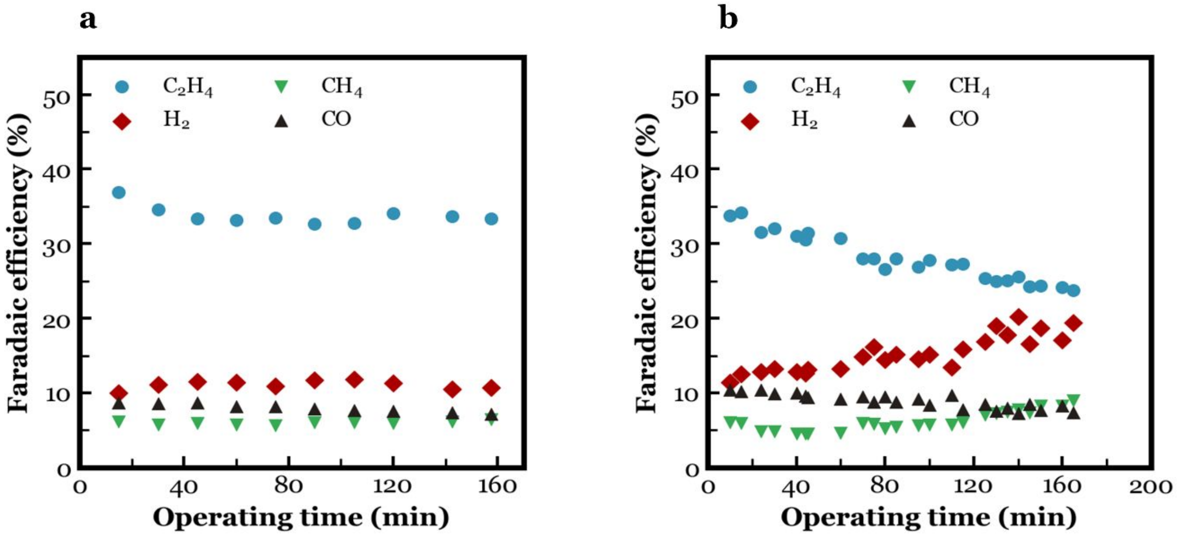

Figure S15. FEs of gas products as a function of operating time during pulsed electrolysis with 15 minutes at -100 mA · cm^{-2} followed by (a) 15 minutes at OCP and (b) 5 minutes at OCP.

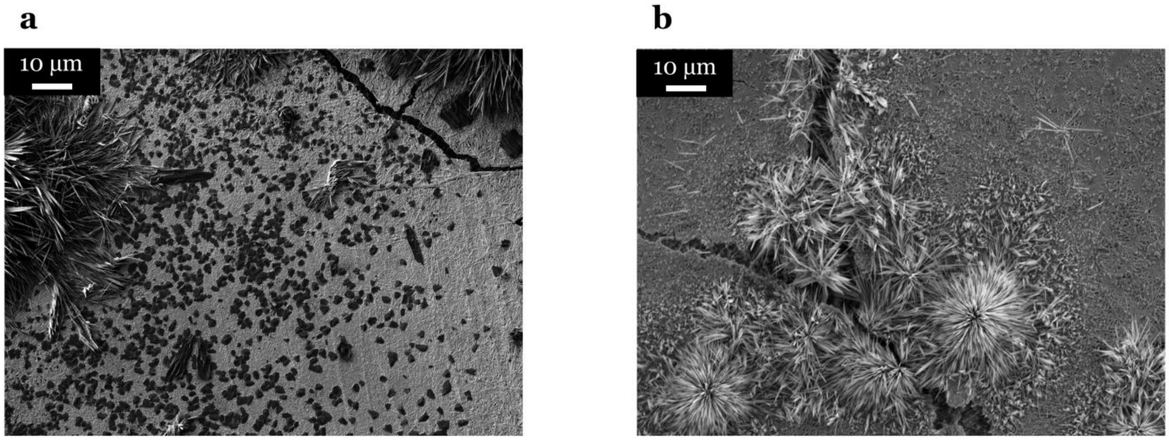

Figure S16. Ex-situ SEM imaging of GDE samples subjected to (a) 15 minutes at -100 mA · cm^{-2} followed by 5 minutes at OCP (1000x magnified, E = 1.0 kV) and (b) 15 minutes 100 mA · cm^{-2} followed by 15 minutes at OCP (1000x magnified, E = 8.0 kV).

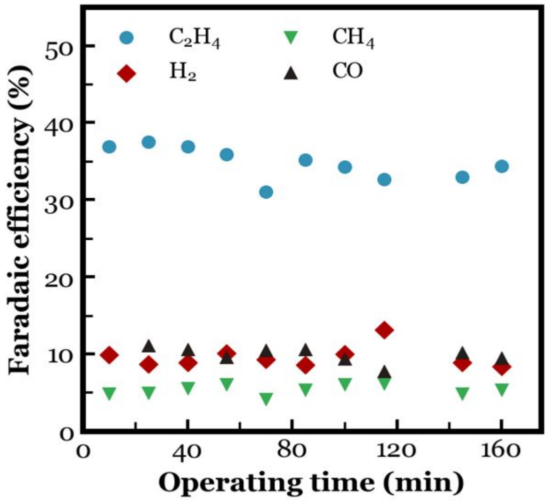

Figure S17. FEs of gas products as a function of operating time for a pulsed operation. 1 hour at -100 mA · cm^{-2} was followed by 15 minutes at OCP.

Table S4. Dissolved Cu mass measured by ICP-MS at different OCP times.

**OCP time (min)**	**Dissolved Cu mass**
3	0.08 mg/L · min
5	0.05 mg/L · min

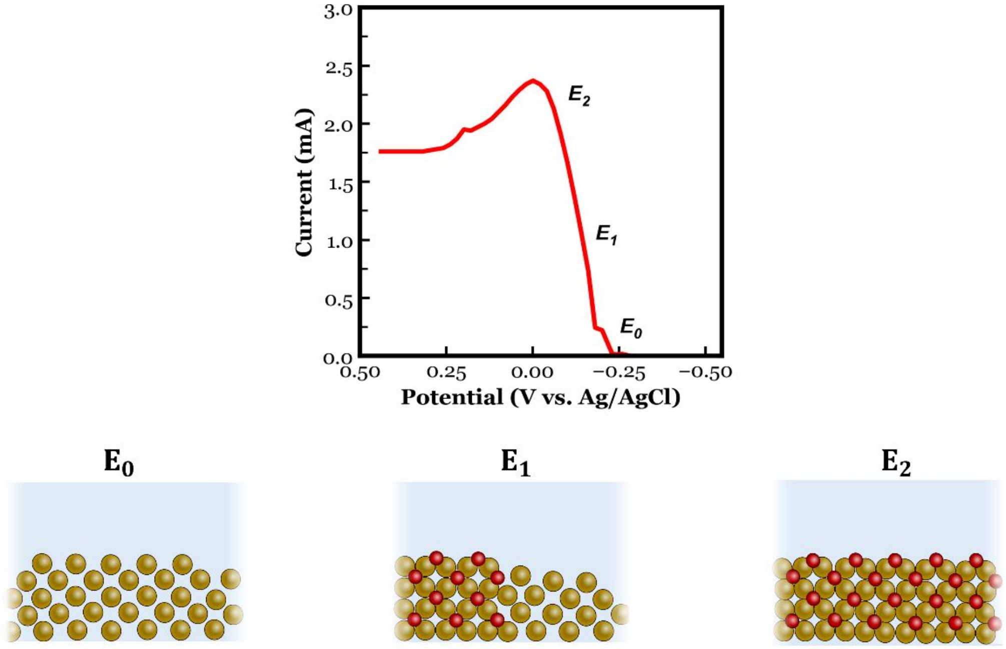

Figure S18. An anodic polarization curve is given for the Cu_2O formation as given by equation 1 in the manuscript. E_0 is the onset potential of Cu_2O formation. This value is very close to that expected from the Pourbaix diagram of Cu. Shifting to more positive potentials results in a higher observed current as the Cu is electrochemically oxidized to Cu_2O (E_1). At E_2, the maximum oxidation current is obtained.

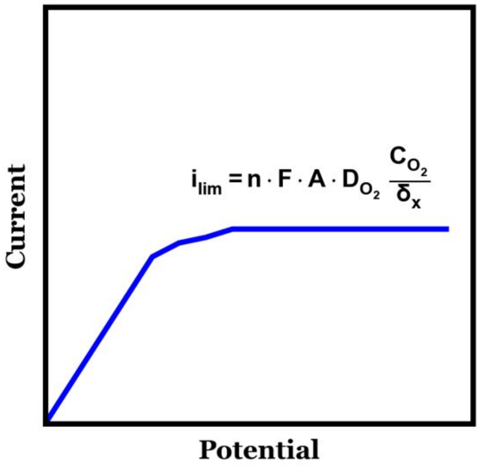

Figure S19. A cathodic polarization curve of the oxygen reduction reaction. Shifting to more cathodic potentials results in a larger current. The limiting reduction current (i_{lim}) is given by the equation

implemented in the plot. As evident from this equation, increasing the oxygen content of the catholyte (C_{O_2}), or decreasing the diffusion distance (δ_x) will allow for a higher limiting current to be achieved.

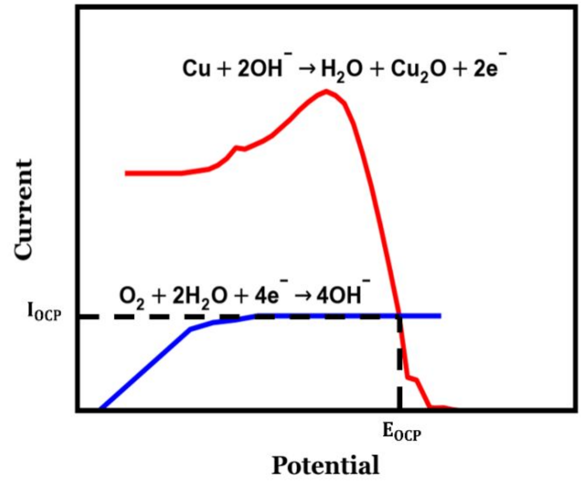

Figure S20. A schematic Evans diagram showing the anodic polarization curve of Cu oxidation to Cu_2O and the cathodic polarization curve of the oxygen reduction reaction. The intersect between the two curves represent at what potential the half reactions generate and consume charge at the same rate.

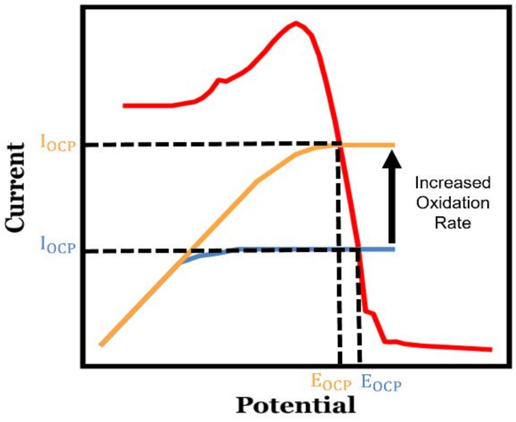

Figure S21. Evans diagram containing the anodic polarization curve of the copper oxidation reaction (red) and the cathodic polarization curve of oxygen reduction reaction for a high cathode pressure of oxygen (yellow) and a low cathode pressure of oxygen (blue).

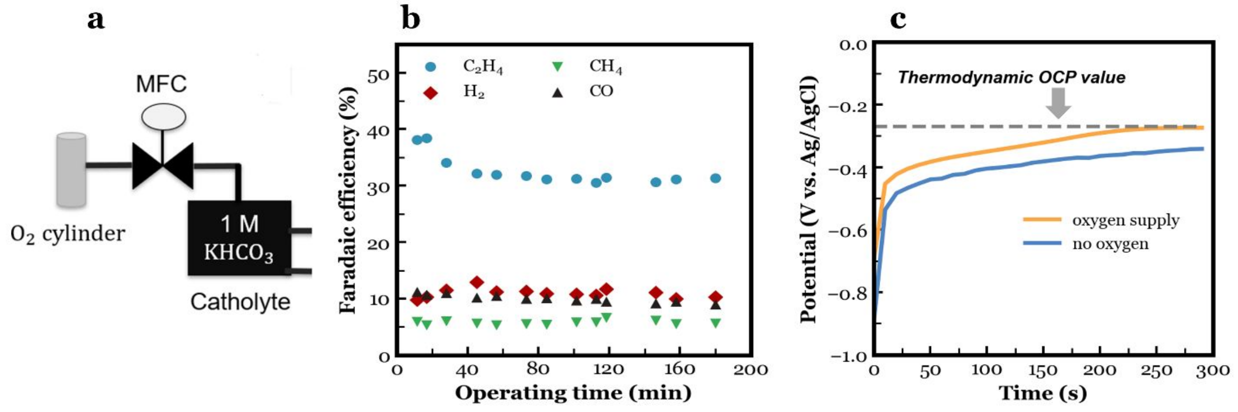

Figure S22. (a) Adaptation made to setup shown in Fig. S2 to supply additional oxygen into the catholyte's headspace. (b) FEs of gas products as a function of operating time with 15 minutes at -100 mA · cm^{-2} and 5 minutes at OCP using the setup adaptation depicted in (a). OCP curves measured during anodic phase for operation with and without additional oxygen supply.

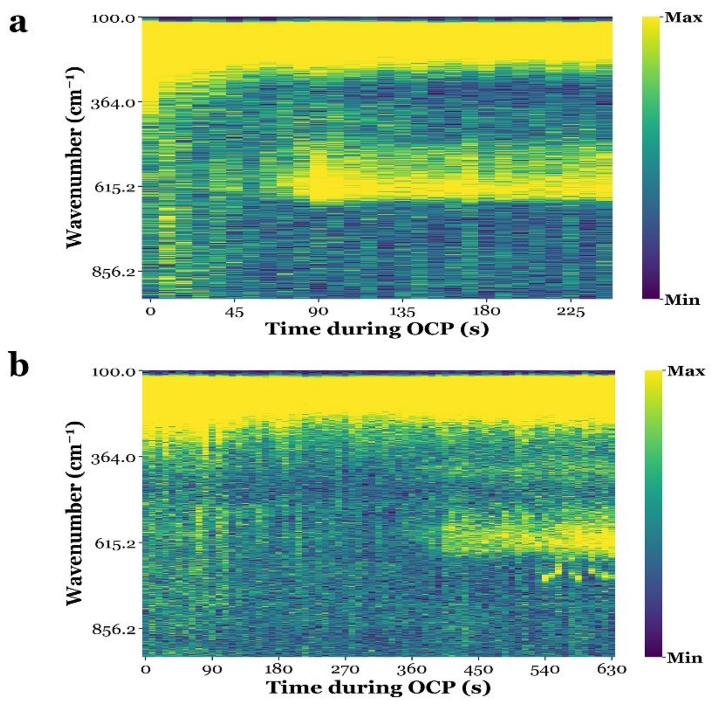

Figure S23. (a) A heatmap expressing the observed Raman signal intensity as a continuous function of wavelength and time during OCP with additional oxygen supply. (b) A heatmap expressing the observed Raman signal intensity as a continuous function of wavelength and time during OCP without additional oxygen supply.

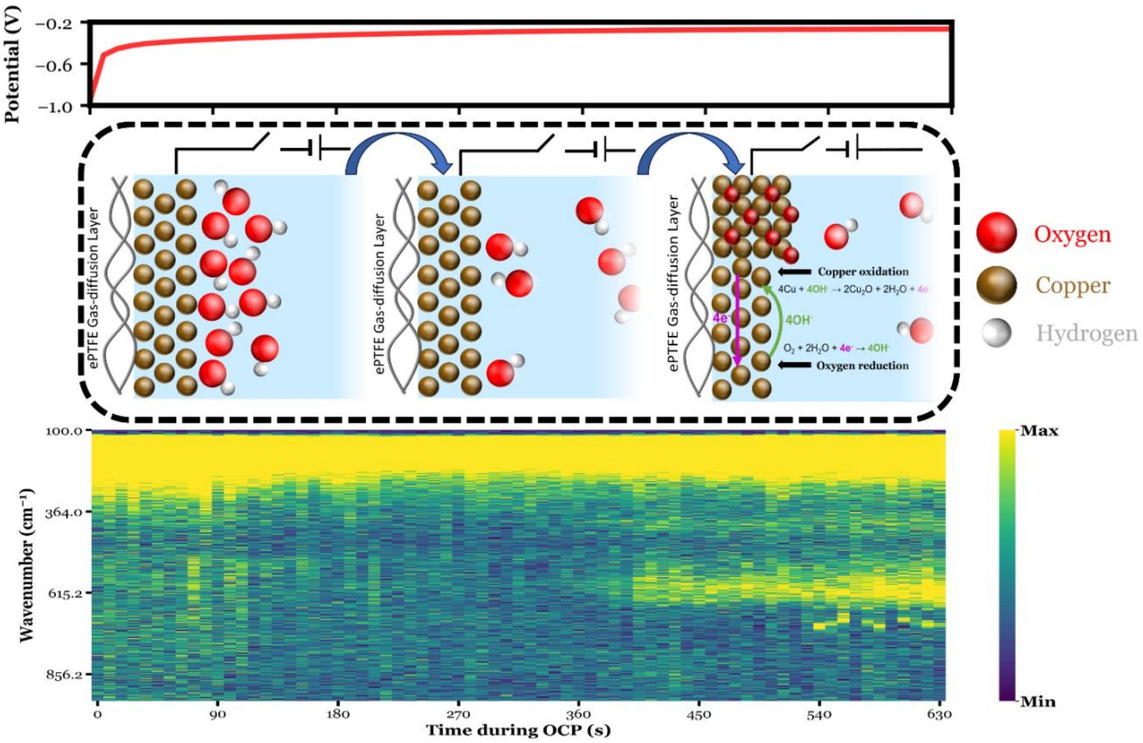

Figure S24. A schematic illustrating the correlation between the monitored OCP curve, local environment and the formation of Cu_2O as visualized by the Raman signal at 630 cm^{-1}.

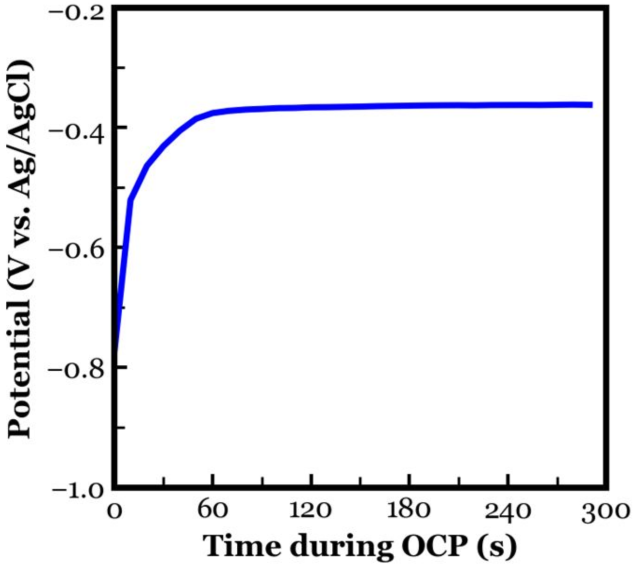

Figure S25. The measured potentials during a 5 minute OCP period after 1 hour at -100 mA · cm^{-2} in 0.5 M K_2CO_3.

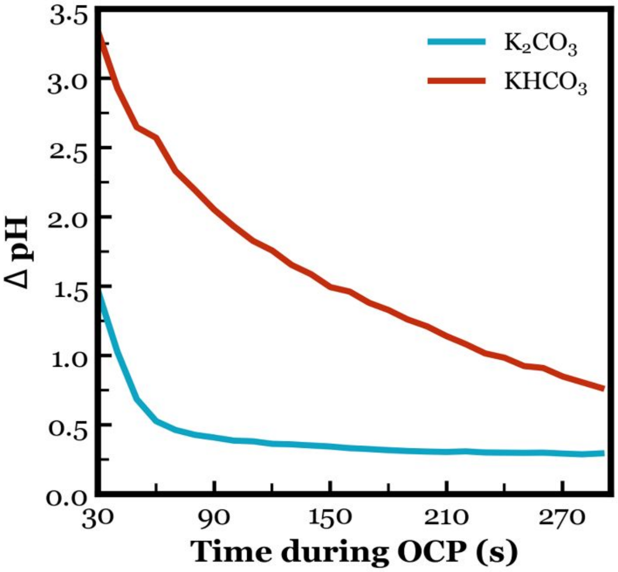

Figure S26. Difference between local pH and bulk pH during OCP time for 0.5 M K_2CO_3 and 1 M $KHCO_3$.

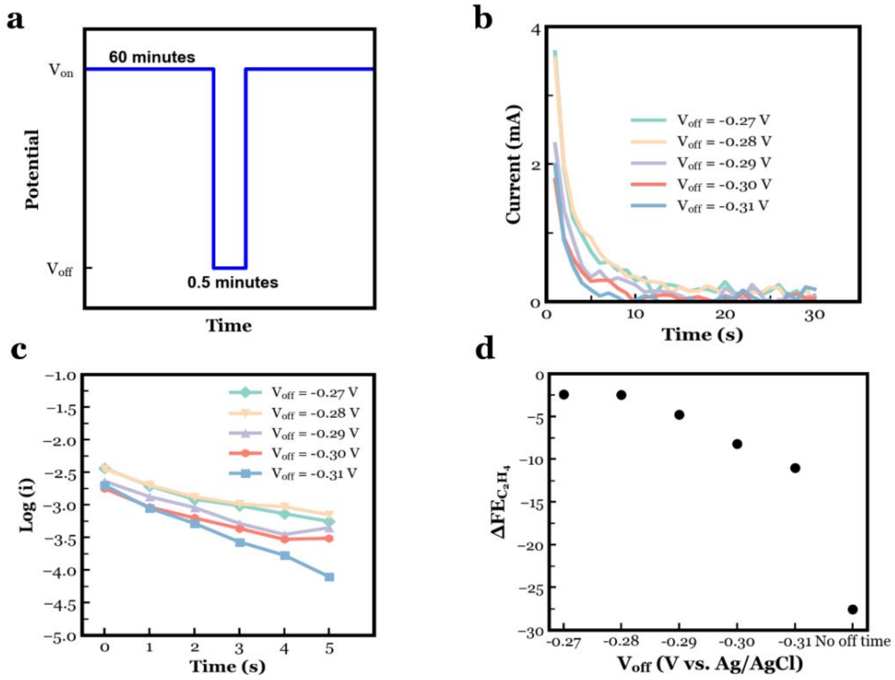

Figure S27. (a) Current curve that was applied to the PEEK-flow cell. 1 hour at a current density of -100 mA · cm^{-2} was followed by 30 seconds at an 'off' potential (V_{off}). (b) Current as a function of time following the potential step. (c) A log(i) vs time linear relationship was found for the first 4 seconds after

switching from the reduction phase to the V_{off} potential. (d) Change in the FE of ethylene as a function of the implemented V_{off} potential after 140 minutes of operating time during pulsed electrolysis.

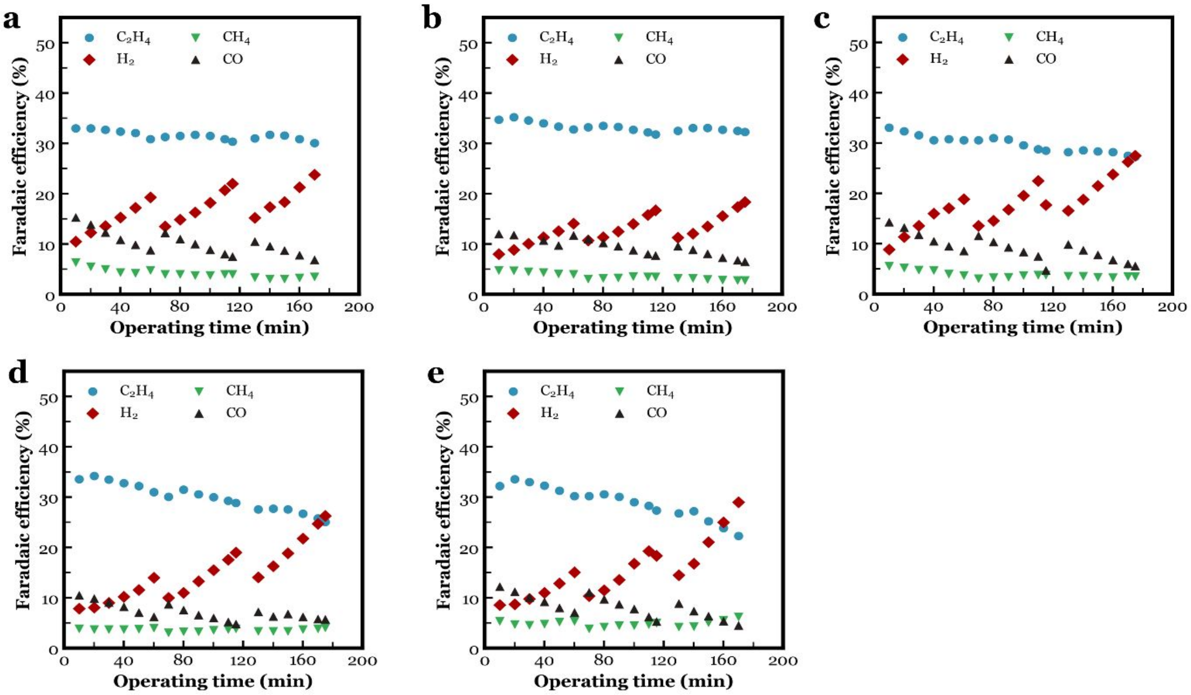

Figure S28. The FEs of gas products as a function of operating time for different V_{off} potential values (vs. Ag/AgCl). (a) V_{off} = -0.27 V. (b) V_{off} = -0.28 V. (c) V_{off} = -0.29 V. (d) V_{off} = -0.30 V. (e) V_{off} = -0.31 V.

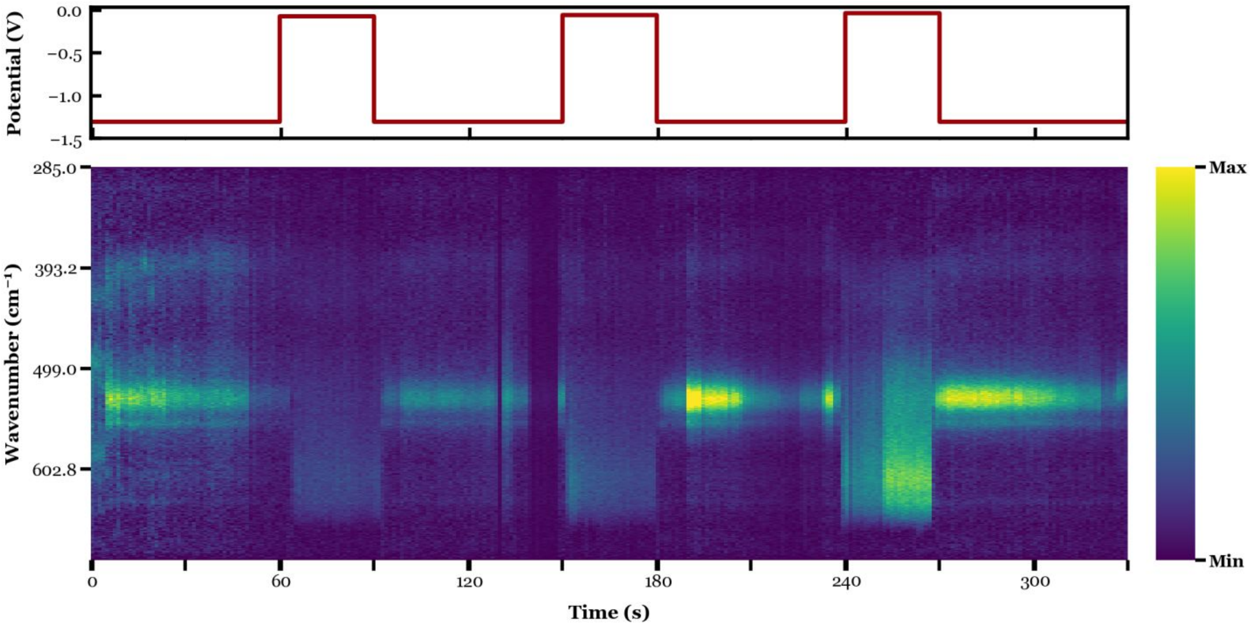

Figure S29. A heatmap expressing the observed Raman signal intensity as a continuous function of wavelength and time. The potential applied during the experiment is plotted as a function of the experimental time above the heatmap. -1.3 V vs. Ag/AgCl was alternated with -0.065 V, -0.05 V, -0.03 V vs. Ag/AgCl, respectively.

The potential applied during the *in situ* Raman measurement is plotted above the heatmap in Fig. S28, showing the distinct reduction and oxidation phases. During every reduction phase, a Raman signal in between 500 to 520 cm^{-1} appears. This corresponds to copper-carbon reaction intermediate complexes as was shown in other scientific works.[3-5] As previously mentioned, Raman signals at 630 cm^{-1} (next to 390 and 520 cm^{-1}) are characteristic for the presence of Cu_2O.[5] Shortly after an oxidation potential was applied, the intensity of the observed signals at this wavenumber increases. With the closed electrical circuit comes, in contrast to chemical oxidation, immediate formation of Cu_2O. Cu_2O on the surface is then also quickly reduced during the subsequent reduction phase.

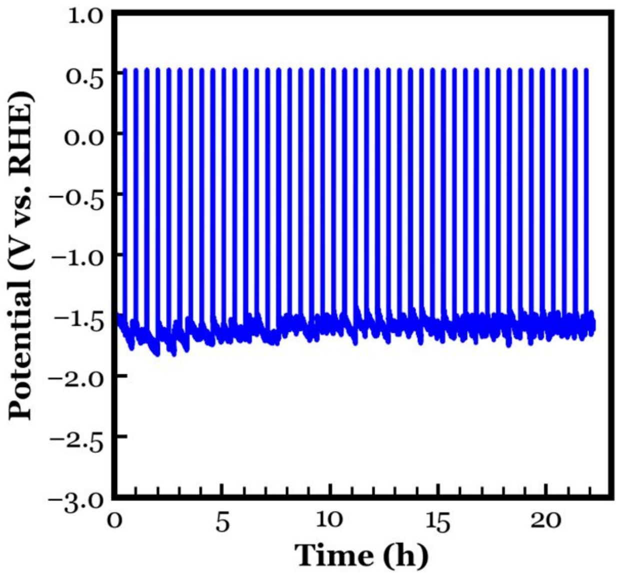

Figure S30. Potential (V vs. RHE) without iR compensation during the stability test as a function of time.

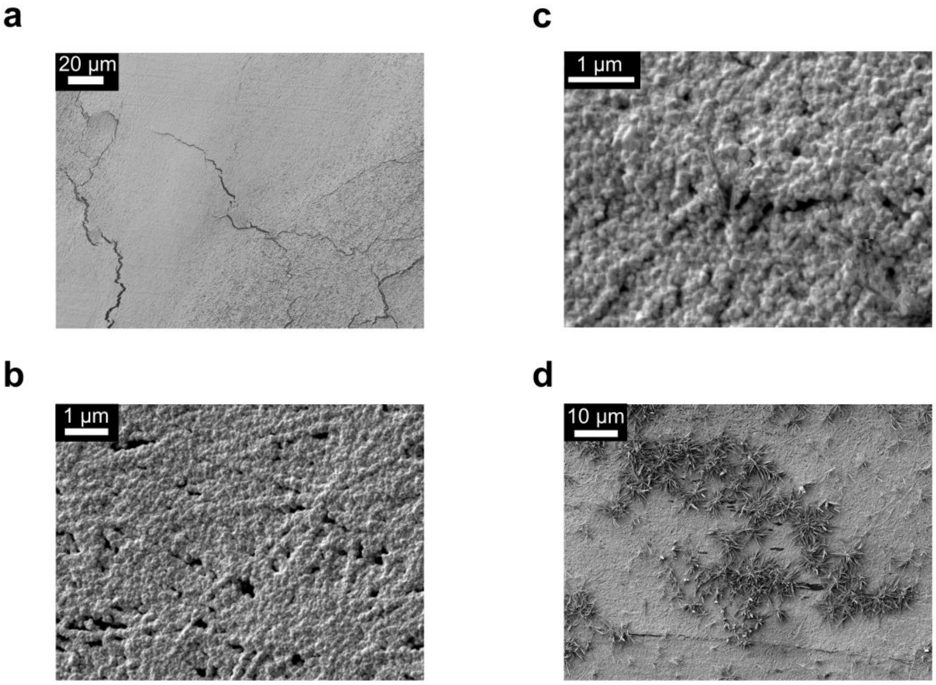

Figure S31. Different SEM images taken of the center (a,b) and the perimeters (c, d) of the Cu GDE after the stability test. (a) 550x magnified, E = 1.0 kV. (b) 14000x magnified, E = 1.0 kV. (c) 23000x magnified, E = 1.0 kV. (d) 1400x magnified, E = 1.0 kV. The present cracks most likely originate from the ex-situ oxidation. Nanowires are $Cu(OH)_2$ formed through cycled copper dissolution and redeposition.

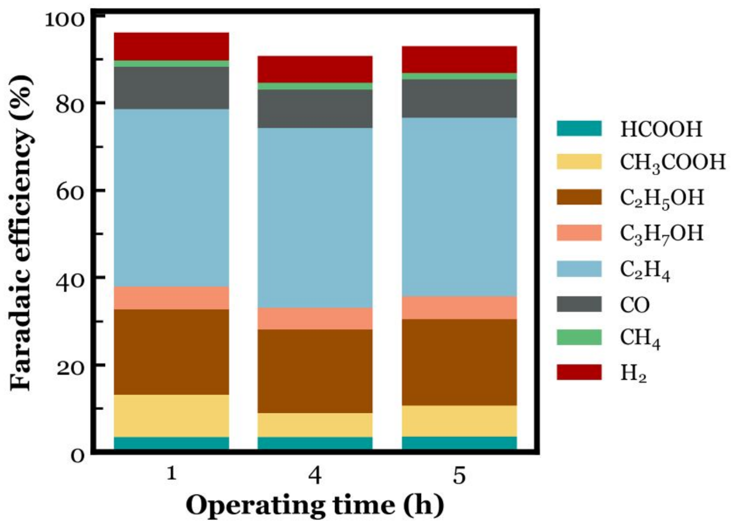

Figure S32. Product distribution at three distinguished operating times during the stability test.

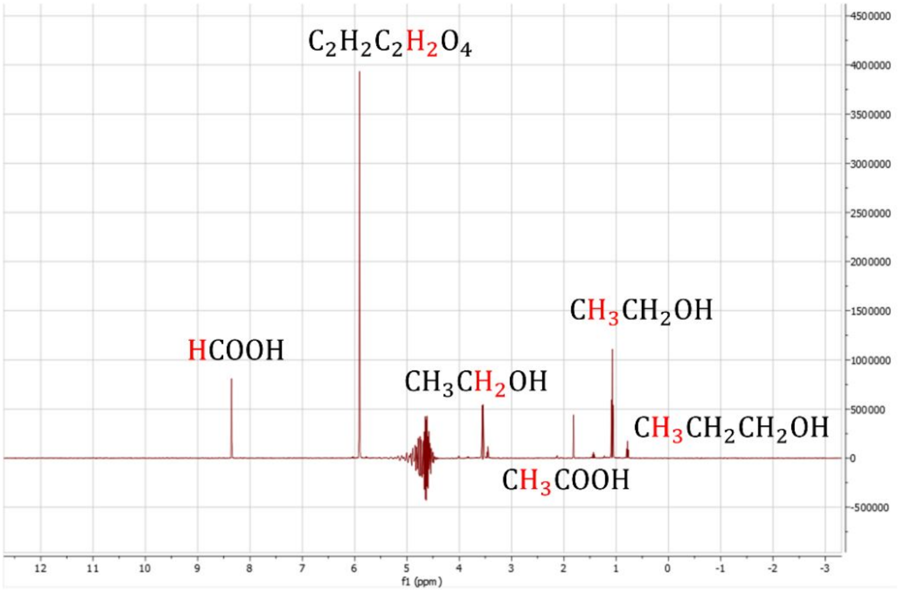

Figure S33. ^{1}H-NMR spectrum (600 MHz, water EIS) of catholyte sample taken during stability test (t = 4 hours). Reference standard used was 40 mM of maleic acid in D_2O.

References

(1) Nguyen, T. N.; Dinh, C.-T. Gas Diffusion Electrode Design for Electrochemical Carbon Dioxide Reduction. Chem. Soc. Rev. 2020, 49 (21), 7488–7504. https://doi.org/10.1039/D0CS00230E

(2) Iglesias Van Montfort, H.-P.; Burdyny, T. Mapping Spatial and Temporal Electrochemical Activity of Water and CO 2 Electrolysis on Gas-Diffusion Electrodes Using Infrared Thermography. ACS Energy Lett. 2022, 7 (8), 2410–2419. https://doi.org/10.1021/acsenergylett.2c00984.

(3) Moradzaman, M.; Mul, G. In Situ Raman Study of Potential-Dependent Surface Adsorbed Carbonate, CO, OH, and C Species on Cu Electrodes During Electrochemical Reduction of CO 2. ChemElectroChem 2021, 8 (8), 1478–1485. https://doi.org/10.1002/celc.202001598.

(4) An, H.; De Ruiter, J.; Wu, L.; Yang, S.; Meirer, F.; Van Der Stam, W.; Weckhuysen, B. M. Spatiotemporal Mapping of Local Heterogeneities during Electrochemical Carbon Dioxide Reduction. JACS Au 2023, 3 (7), 1890–1901. https://doi.org/10.1021/jacsau.3c00129.

(5) De Ruiter, J.; An, H.; Wu, L.; Gijsberg, Z.; Yang, S.; Hartman, T.; Weckhuysen, B. M.; Van Der Stam, W. Probing the Dynamics of Low-Overpotential CO_2-to-CO Activation on Copper Electrodes with Time-Resolved Raman Spectroscopy. *J. Am. Chem. Soc.* **2022**, *144* (33), 15047–15058. https://doi.org/10.1021/jacs.2c03172
